# Universal cryogenic transfer of liquid metal particles in polymers for wafer-scale stretchable integrated electronics

**DOI:** 10.1038/s41467-026-70101-2

**Published:** 2026-02-26

**Authors:** Do Hoon Lee, Seungkyu Lee, Minyong Park, Junehyeok Kim, Hanbit Jin, Su Yeong Kim, Donghyun Lee, Young-Soo Lim, Jun Chang Yang, Taehoon Lee, Byungkook Oh, Sang Yu Sun, Do-Wan Kim, Sihong Wang, Sung Gap Im, Hye Jin Kim, Sung-Min Park, Jihan Kim, Yang-Kyu Choi, Steve Park

**Affiliations:** 1 School of Electrical Engineering, Korea Advanced Institute of Science and Technology (KAIST), Daejeon, Republic of Korea; 2 Department of Materials Science and Engineering, Korea Advanced Institute of Science and Technology (KAIST), Daejeon, Republic of Korea; 3 Pritzker School of Molecular Engineering, University of Chicago, Chicago, IL USA; 4 Department of Chemical and Biomolecular Engineering, Korea Advanced Institute of Science and Technology (KAIST), Daejeon, Republic of Korea; 5 Electronics and Telecommunications Research Institute (ETRI), Daejeon, Republic of Korea; 6 Center for Advanced Biomolecular Recognition, Korea Institute of Science and Technology (KIST), Seoul, Republic of Korea; 7 NAVER Corporation, Bundang, Republic of Korea; 8 Department of Convergence IT Engineering (CiTE), Pohang University of Science and Technology (POSTECH), Gyeongsangbuk-do, Republic of Korea; 9 Smart 3D Printing Research Team, Korea Electrotechnology Research Institute (KERI), Changwon, Korea; 10 KAIST Institute for Health Science and Technology, Daejeon, Republic of Korea

**Keywords:** Soft materials, Electronic devices

## Abstract

Gallium-based liquid metals (LMs) are promising materials for stretchable electronics due to their metallic conductivity and deformability. However, the fabrication of large-area stretchable integrated electronics using LMs on various polymers remains challenging due to their high surface tension, fluidity, and poor wettability. Current techniques, such as selective wetting and lift-off processes, face limitations related to substrate compatibility and Ga/metal alloying, hindering their applicability in integrated electronic systems. To address these challenges, we developed a high-resolution top-down etching-based photolithography combined with a universal cryogenic transfer method for transferring patterned LM particles (LMPs) in various polymer substrates. The cryogenic environment modifies the interfacial bonding between the LMPs and substrates, resulting in a universal transfer. The resulting liquid metal particle network embedded polymer (LNEP) exhibits high electrical conductivity (~1.71 × 10⁶ S/m), stability, and strain-insensitive performance across various polymers. This process is scalable to large-area fabrication, overcoming the limitations of existing LM patterning techniques. Leveraging this approach, we demonstrated the use of LNEP ranging from skin-conformal wearable sensors to hybrid stretchable circuits and implantable devices, demonstrating the universality of the method. This technique establishes a scalable pathway for stretchable electronics in advanced applications.

## Abstract (web summary)

Stretchable liquid-metal electronics is limited by high surface tension, fluidity, and poor wettability. Here, Lee et. al. presents a universal cryogenic transfer method for liquid metal particles, enabling high-throughput fabrication of wafer-scale stretchable integrated electronics with robust electrical performance.

## Introduction

Stretchable electronics have gained significant attention due to their wide range of potential use in healthcare^1^, wearable devices^2,3^, and augmented and virtual reality (AR/VR)^4,5^. To realize stretchable electronics, intrinsically stretchable conductors such as conductive polymers^6,7^, conductive nanocomposites^8,9^ and gallium-based liquid metals (LMs) have been reported. Among these, gallium-based LMs have recently been highlighted for their metallic conductivity, exceptional deformability, and biocompatibility^10,11^. However, to enable the versatile use of LM-based stretchable electronics in commercial applications, it is essential to achieve high-resolution patternability over large-areas, high-throughput, electrical/mechanical stability, and universal compatibility with various polymer substrates. To satisfy these criteria, various LM patterning methods have been developed^12^, including laser ablation^13^, nozzle printing^14^, screen printing^15^, vacuum filling^16^, transfer method^17^, and photopatterning method^18–22^. However, these methods often suffer from critical trade-offs, for example, laser ablation and nozzle printing achieve high resolution but make it difficult to achieve high-throughput, while stencil printing and transfer methods are scalable but offer poor resolution or have substrate limitations. Among these approaches, photopatterning has high potential for commercial use as it is already a well-established standard process, thus making it easy for industrial implementation. It also has technologically critical attributes such as high-resolution, high-throughput, high-pattern fidelity, and scalability to large-area (Supplementary Table 1),

Photopatterning methods of LMs have been realized with various approaches: (1) selective wetting of LM on patterned metal^18,19^, (2) the lift-off process^20,21^, and (3) mixing LM particles (LMPs) with UV-curable polymers^22^. While these methods offer the aforementioned advantages of photopatterning, each has its limitations: (1) selective wetting of LM on patterned metal is constrained by two factors: a strong correlation between the LM film thickness and the pattern pitch, and inevitable Ga/metal alloying, which changes electromechanical properties^23,24^, (2) the lift-off process is limited to specific substrates due to the compatibility issues with the developer and organic solvents, and (3) mixing LMPs with UV-curable polymers is also restricted to certain substrates because of their wetting properties. Moreover, all of these methods suffer from LM leakage under mechanical stimuli and face difficulties in forming robust interfaces with rigid electronic chips. To address these limitations, Lee et al. have introduced utilizing acoustic fields to assemble a network of LMPs within various polymer matrices^25^. However, this method is not suitable for mass production processes as it is challenging to achieve large-area uniformity and high throughput, while the photolithographically patterned structures cannot be stretched. Therefore, to fabricate broadly applicable LM-based integrated stretchable electronics, there is still a demand for a patterning method that is universal across various polymers, electrically and mechanically stable, achieves high-yield over a large-area, and ensures seamless integration with rigid electronic chips.

**Fig. 1:**
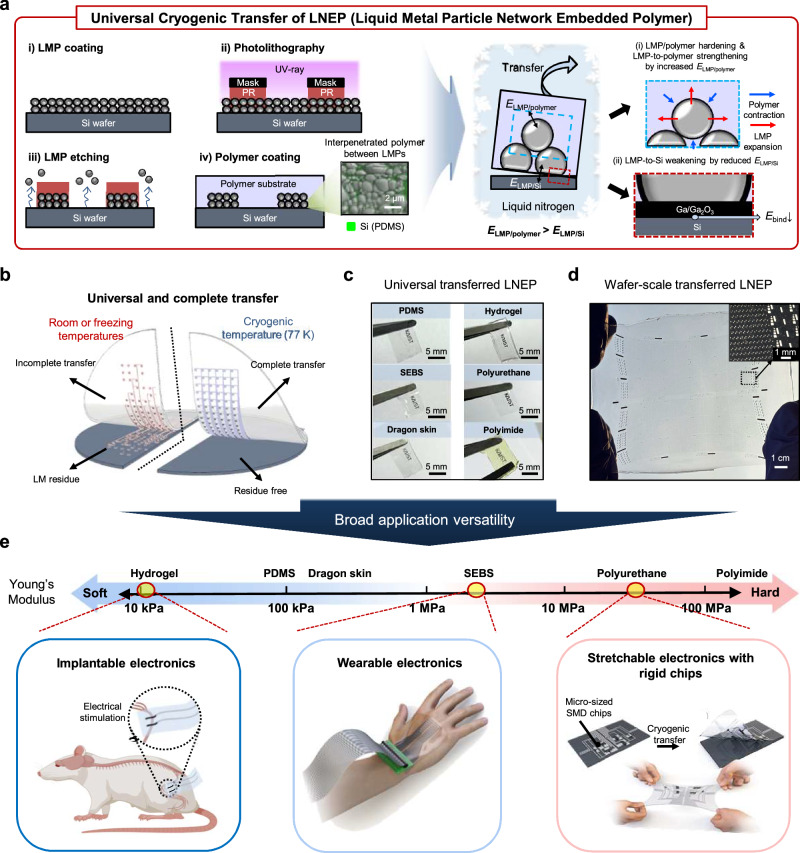
LNEP for large-area stretchable integrated electronics. **a** Schematic illustrations of the patterning (left) and cryogenic transfer process (right) for LMPs. The inset SEM image shows interpenetrated polymer matrix between LMPs after the polymer coating. **b** A comparison of complete cryogenic transfer (77 K) with incomplete room or freezing temperatures transfer. **c** Photographs of the transferred LNEP films on various polymer substrates. **d** Wafer-scale transferred LNEP film using the cryogenic transfer method. **e** Demonstrating the broad application versatility of LNEP across polymer matrices with various Young’s moduli. Rat figure of (**e**) was created in BioRender. Rachim, V. (2025) https://BioRender.com/xd13cby.

Here, we report a universal high-yield and high-resolution approach to transfer LMP patterns in various polymer matrices over large-areas (Fig. 1). LMP lines were first patterned on a silicon (Si) substrate (i.e., donor substrate) through standard photolithography and a wet-etching process (Fig. 1a, left (i-iii)). Subsequently, a polymer substrate (i.e., acceptor substrate) was coated onto the LMP patterns (iv). The coated polymer matrix interpenetrated between LMPs, as shown in the EDS-SEM image (Fig. 1a, middle). LMP patterns were then transferred to the polymer substrate by cryogenic transfer at 77 K (Fig. 1a, right). As the temperature decreased to 77 K, the interpenetrated polymer matrix and LMP hardened due to glass transition and LMP solidification, leading to interface strengthening induced by volume changes. Meanwhile, the LMP/Si interface became relatively weakened at the cryogenic temperature. The resulting transferred LMP forms a fully percolated liquid metal particle network embedded in polymer (LNEP). This embedding ensures electrical and mechanical stability, contrary to placement on the surface of polymers.

Unlike transfers performed at room or freezing temperatures, cryogenic transfer enables complete and residue-free transfer of LMP patterns onto various polymer substrates (Fig. 1b, c), overcoming the limitations of existing methods (see Supplementary Table 1, 2 for detailed comparison to previous LM patterning and transfer techniques). Figure 1d shows that the cryogenic transfer process is applicable on a wafer scale. On various substrates and resolutions, the transferred LNEP shows a high electrical conductivity of 1.71 × 10^6 ^S/m, and strain-insensitive properties.

By leveraging this universal fabrication method, we implemented broad applications by selecting suitable polymer substrates for each purpose (Fig. 1e). For bioelectronics applications, patterned LMP in tissue-like biocompatible hydrogel was used for monitoring electromyography (EMG) signal and stimulating peripheral nerves. To achieve a skin-like modulus and stretchability, styrene–ethylene–butylene–styrene (SEBS) polymer was applied to a large-area on-skin sensor array, facilitating the development of tactile recognition systems. Finally, tough polyurethane (PU) polymer was used to integrate rigid electronic chips with patterned LMP. These rigid chips can be monolithically transferred along with LMP lines in a one-step cryogenic transfer process. As the polymer matrices embed both the chips and LMP lines, the transferred rigid chips exhibit excellent mechanical robustness when subjected to strain.

## Results

We adopted a photolithography-based wet-etching method to form high-resolution LMP patterns on a Si wafer, focusing on achieving a high-yield, high-pattern fidelity, and large-area uniformity. LMP ink was prepared by sonicating bulk EGaIn and acetic acid in ethyl acetate as a solvent to break bulk EGaIn into micrometer-sized LMPs (full fabrication process is described in detail in the Methods and Supplementary Fig. 1). To attain high-resolution patterns, we limited the maximum size of the LMPs to under 3 μm by using a relatively long sonication time^26^. Given that, nanometer-sized LMPs exhibit solid-like rigidity, we optimized the sonication time to 5 min, yielding an average LMP size of 1.14 μm^27^ (Supplementary Fig. 2).

**Fig. 2:**
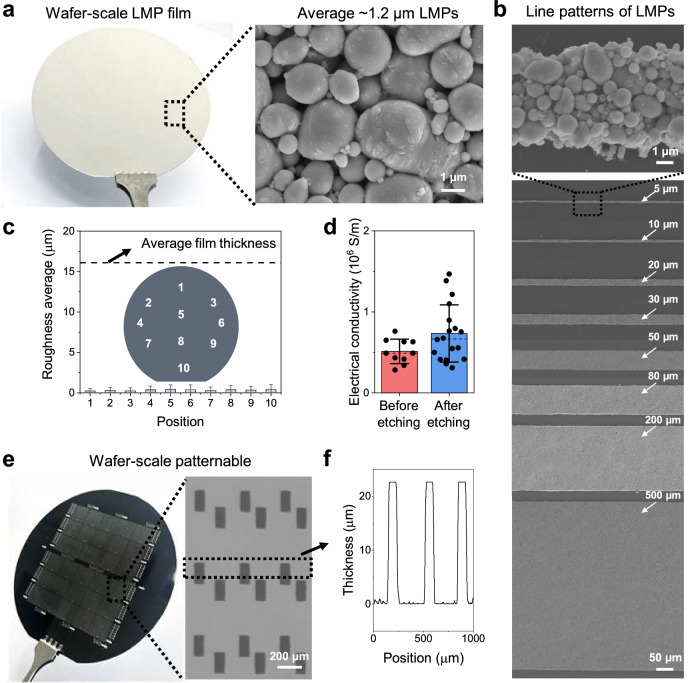
Wafer-scale photopatterning of LMPs. **a** Photograph of the wafer-scale LMP film and magnified SEM image of the film. **b** Top view SEM images of patterned LMP lines with various line widths ranging from 5 μm to 500 μm. **c** Surface roughness of coated LMP film at each position on a 4-inch wafer. The dashed line indicates the average thickness of the film, while the bars present the roughness at each position. **d** Electrical conductivity of the LMP lines before and after the wet-etching process. **e** Photograph of a wafer-scale patterned LMP film (left) and magnified optical microscope image of the patterned LMP (right). **f** The measured height profiles of the fabricated LMP patterns.

To facilitate the top-down lithography of the LMPs, a uniform coating of the LMP film is essential. Therefore, we undertook the spin-coating of the LMPs instead of the widely used spray-coating^28,29^ or drop-casting techniques^30^, which result in non-uniform films. We adopted the ethyl acetate with excellent wettability on Si and controlled the evaporation kinetics to ensure uniform deposition, thereby avoiding the coffee-ring effect and cracking^31,32^ (Fig. 2a, c and Supplementary Figs. 3–5). Additionally, we were able to control the final thickness of the LMP film by adjusting the LM content in the ink, the spin-coating speed, and the number of coatings (Supplementary Fig. 6). Next, we coated and patterned a positive photoresist on the LMP film, followed by a wet-etch process to create fine patterns (Supplementary Fig. 7). The wet-etching of LMPs has not been reported, likely due to the difficulty in controlling the etching selectivity and achieving uniform etch rates. We are able to realize wet-etching through the optimization of both the etchant solution and the processing conditions. Consequently, LMP line width as narrow as 5 μm was achieved with high-pattern fidelity (Fig. 2b and Supplementary Fig. 8). Our LMP films exhibit an average initial electrical conductivity of a 0.512 × 10^6^ S/m resulting from the partial removal of the surface oxide of LMPs by the acidic solvent, and capillary bridging between the LMPs^27,30^. The conductivity was further increased to an average of 0.733 × 10^6^ S/m during the etching process due to the influence of etchant vapors^33^ (Fig. 2d and Supplementary Fig. 9). The conductivity increased to 1.71 × 10^6^ S/m after transferring the LMP film to the polymer and activating it (details to be explained below). Figure 2e shows the patterned LMP film on a 4-inch Si wafer. As shown in the 3D profiles in Fig. 2f, the patterned LMP lines were cleanly etched and maintained a uniform thickness.

After patterning the LMP film on the Si substrate, a transfer process is needed to integrate the LMP into soft electronics. To achieve universal applicability, the transfer method should be capable of transferring the entire pattern to various polymer substrates with a high-yield. Thus, we propose a cryogenic transfer process to transfer LMPs to polymer substrates by utilizing the changes in their mechanical and chemical properties at low temperatures.

**Fig. 3:**
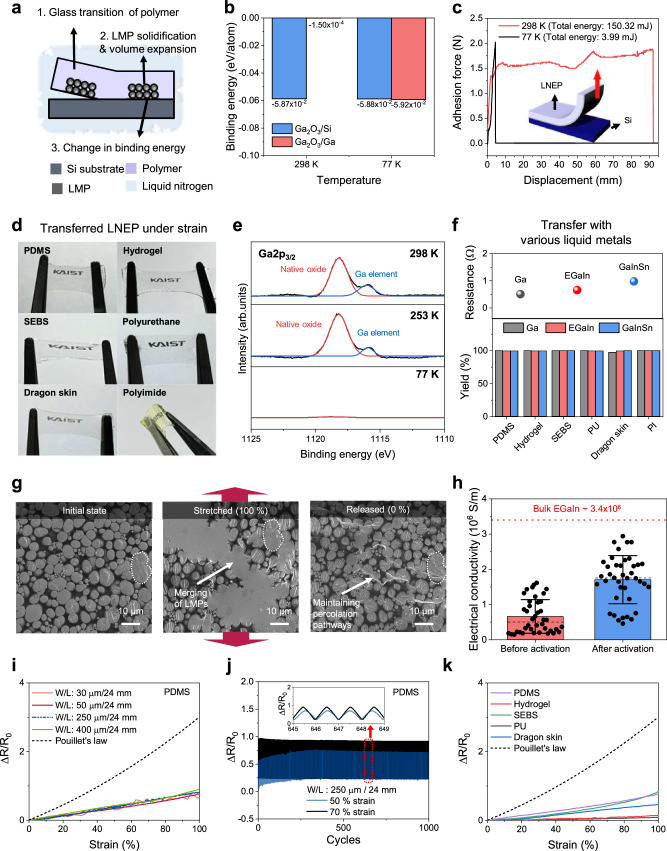
Universal cryogenic transfer. **a** A schematic illustration of the cryogenic transfer mechanism. **b** Calculated binding energy between Ga_2_O_3_/Si and Ga_2_O_3_/Ga interface under different temperatures (298 K, 77 K). **c** Adhesion forces between Si-LNEP obtained by 90° peeling tests under different temperatures (298 K, 77 K). **d** Photograph of transferred LNEP on various polymer substrates. **e** XPS spectra of initial wafer after transfer at different temperatures (298 K, 253 K, 77 K). **f** Cryogenic transfer to polymer substrates using various liquid metals (Ga, EGaIn, GaInSn) and the yield of the transfer process. **g** SEM images showing LNEP in the initial state, 100% stretched state, and released state. The percolation pathways were maintained even in the released state. **h** Comparison of the electrical conductivity of LNEP before and after activation. **i**, The resistance response of LNEP in PDMS with different widths (30, 50, 250, 400 μm) at the same length of 24 mm and theoretical prediction based on Pouillet’s law. **j** The resistance responses of LNEP lines in PDMS under 50% and 70% strain during 1000 cycles, with a width of 250 μm and length of 24 mm, showing high long-term stability. The inset graph shows a magnified view of the cyclic test. **k** The resistance responses of LNEP in various polymers (PDMS, Hydrogel, SEBS, PU, Dragon skin, PI).

To transfer the LMP film, we first spin-coated various polymer films onto the LMP-patterned Si substrate, followed by thermal curing of the polymer films (details of the polymer solution fabrication process are provided in the Methods). Subsequently, the substrate was immersed in liquid nitrogen to lower the temperature below glass transition temperature (*T*_g_) of polymers (Supplementary Fig. 10 and Supplementary Table 3). Under the cryogenic condition, the detachment of the LMP patterns from Si occurred due to (1) the glass transition of the polymer^34,35^, (2) the solidification^36^ and volumetric expansion of the LMPs^37,38^, and (3) changes in the binding energy at the Ga_2_O_3_/Ga and Ga_2_O_3_/Si interfaces^39,40^ (Fig. 3a). Polymers undergo a glass transition as the temperature drops below *T*_g_, leading to volumetric contraction and weakening the binding between the polymer and Si. Simultaneously, LMP solidification and ~2% volumetric expansion occur, enhancing the binding between the LMPs and the polymer matrices^41^ (Supplementary Video 1). In addition, the interfacial binding energy of the bottom-most LMP layer plays a crucial role in ensuring complete detachment. To elucidate the cryogenic transfer mechanism at the bottom-most LMP layer, we conducted ab initio molecular dynamics (AIMD) simulations to calculate the interfacial binding energy between the LMP and the Si wafer at different temperatures. Prior to the AIMD simulations, X-ray diffraction analyses were conducted at different temperatures (298 K, 77 K) to characterize the crystal phase and crystallinity (Supplementary Fig. 11). At room temperature, the LMP consists of a poorly crystallized α-Ga_2_O_3_ shell with a liquid metal core^42^. As the temperature decreases to 77 K, the liquid metal crystallizes into a β-Ga dominant structure while retaining the α-Ga_2_O_3_ shell. With this crystal structure, we conducted AIMD simulation at 298 K and 77 K (Fig. 3b and Supplementary Fig. 12). At 298 K, the binding energy at the Ga_2_O_3_/Ga interface (−1.50 × 10^−4 ^eV/atom) is significantly lower than that at the Ga_2_O_3_/Si interface (−5.87 × 10^−2 ^eV/atom). In contrast, at 77 K, the binding energy at the Ga_2_O_3_/Ga interface (−5.92 × 10^−2 ^eV/atom) exceeds that at the Ga_2_O_3_/Si interface (−5.88 × 10^−2 ^eV/atom), indicating that detachment of the LMP from the substrate becomes significantly easier under the cryogenic condition. The substantial change in the adhesive force at 77 K was further confirmed by 90° peel tests (Fig. 3c). The total energy required to detach the entire film from the Si wafer was measured to be only 3.99 mJ at 77 K, which is significantly lower than the energy required at 298 K (150.32 mJ).

Using the aforementioned cryogenic transfer process, we successfully transferred LMP patterns to various polymer substrates, achieving strong adhesion with the substrates under strain (Fig. 3d). Compared to the LMP-coating method, LNEP demonstrates a robust interface by embedding within the polymer, even on substrates with poor adhesion to LMPs or those prone to swelling during a solution process, such as hydrogel (Supplementary Fig. 13). Also, LNEP consists of densely packed liquid metal particles that maintain a percolation network and uniform thickness after being transferred (Supplementary Figs. 14, 15a, b). In contrast, at room temperature (298 K), the LMP patterns remain on the initial wafer due to an incomplete transfer (Supplementary Fig. 16), resulting in unfilled areas on the transferred substrate and a recessed surface morphology (Supplementary Fig. 15c). Additionally, certain defects such as voids may form as a consequence of the incomplete transfer (Supplementary Fig. 15d).

The completeness of the cryogenic transfer was verified through X-ray photoelectron spectroscopy (XPS) performed on the initial wafer after transfers at different temperatures (298 K, 253 K, and 77 K) (Fig. 3e and Supplementary Fig. 17). When transferring at moderate temperatures (298 K and 253 K), peaks corresponding to the native Ga oxide, Ga, and In elements were observed, indicating the presence of LMP residues remaining on the initial Si wafer after the transfer. In contrast, following the cryogenic transfer, only a few native oxide peaks were observed on the initial wafer, confirming the effectiveness of the process^43^.

Our cryogenic transfer method is applicable to various polymers and various types of liquid metals. We successfully formed LNEP by transferring Ga, EGaIn, and Galinstan onto 6 polymer substrates with varying mechanical properties, achieving a high-yield (Fig. 3f and Supplementary Figs. 18–21). We also demonstrated that the transfer process is feasible at wafer-scale, ensuring consistent performance and reliability across large areas (Supplementary Fig. 22).

Using LNEP in polymer substrates, we measured the electrical properties under deformation to demonstrate the potential of our film as stretchable electrodes. Previously reported incorporation of LM in elastic printed circuit boards involved coating LMP^14,22^ or bulk LM^44^ on polymer substrates. However, the coated bulk LM undergoes surface oxidation and delamination due to the poor adhesion between the LM and polymer substrate, leading to a rapid increase in the resistance during repeated strain tests (Supplementary Fig. 23a). Similarly, the coated LMP undergoes an electrical failure due to crack generation on the film surface when stretched (Supplementary Fig. 23b). Hence, LMP-embedded polymer composites are widely used to utilize LM in various polymers^45^. These composites are fabricated by mixing LMPs with the polymer, resulting in a few micrometers thick insulating polymer matrix between the LMPs. Consequently, even after repeated stretching and releasing, incomplete percolation pathways are formed (Supplementary Fig. 24a), resulting in poor conductivity^46^. SEM images confirmed the structure of the embedded-LMP polymer composites in its initial, stretched, and released states, with the red box inset in the stretched state showing a few micrometers of insulating polymer matrix between the LMPs (Supplementary Fig. 24b).

The cryogenic transfer method, on the other hand, transfers aggregated LMP patterns into the polymer matrix, allowing the polymer to fill the gaps between the LMPs (Supplementary Fig. 25a) while maintaining fully connected electrical pathways between the LMPs. This unique structure of LNEP overcomes the critical limitations of the coating and composite forming methods, providing both high mechanical stability and electrical conductivity while simultaneously possessing the high-resolution patternability over large areas.

SEM images confirm that when the LNEP is stretched, the LMPs break and aggregate, forming electrical connections (Fig. 3g, left, middle)^47^. After releasing the strain, the LMPs return to an arrangement similar to the initial state, however, with the LMPs electrically connected (Fig. 3g, right) to form percolating pathways. This activation process also occurs even in micro-patterned LNEP (Supplementary Fig. 25b). This behavior is attributed to the rupturing of the adjacent oxide shells upon stretching, thus merging of the particles. Because the interpenetrated polymer matrix anchors the LMPs, the LMPs’ original configuration is maintained even after the strain is released (Supplementary Fig. 25a, c).

To understand the rupturing of the oxide shell on the stretched LNEP, a Finite Element (FE) simulation was conducted (Supplementary Fig. 26). As the distance between the LMPs decreases to less than nanometers, the stress at the interface increases sharply. Given that the native oxide is inherently brittle, it is prone to breaking under an external impact or stress. The average electrical conductivity of cryogenically transferred LNEP before activation is 0.662 × 10^6^ S/m, similar to the electrical conductivity after the etching process. After activation, it increases to 1.71 × 10^6^ S/m, about half the value of the bulk LM (Fig. 3h). The relative resistance change (Δ*R/R*_0_) of LNEP lines under strain was measured across different widths (30, 50, 250, 400 µm) (Fig. 3i and Supplementary Fig. 27). LNEP lines in PDMS showed only a 90% increase in resistance after being stretched to 100%, compared to the 300% increase in the bulk conductors, as described by Pouillet’s law [$\triangle$*R/R*_0_ = (1+*ε*)^2^ − 1] (Supplementary Fig. 28). The lower gauge factor of the LNEP patterns can be explained as sustained percolation between the LMPs^48^. Similar results were attained for LNEP in SEBS (Supplementary Fig. 29).

LNEP can also be utilized to fabricate stretchable vertical interconnect accesses (VIAs), offering a significant advantage for multilayer stretchable electronic applications. LNEP-based VIAs exhibit strain-insensitive properties under mechanical deformation, ensuring stable electrical connections between layers (Supplementary Fig. 30).

Cyclic tests were conducted to confirm the electrical stability of LNEP under repeated strains. Because LNEP maintains its percolation pathways, it exhibited stable and consistent resistance changes during cycling (Fig. 3j and Supplementary Fig. 31) and long-term stability over 6 months (Supplementary Fig. 32). To analyze the mechanical stability of the LNEP lines further, we measured their resistance under various mechanical deformations, such as twisting and compression (Supplementary Fig. 33). The LNEP lines showed stable electrical conductivity, even when twisted up to 675°, and subjected to cyclic compression with 100% strain for 1000 cycles.

In addition, to confirm the universal applicability of the cryogenic transfer, the electromechanical properties of LNEP in various polymers (PDMS, Hydrogel, SEBS, PU, Dragon skin) were characterized (Fig. 3k). A relatively low gauge factor was observed for LNEP lines in all polymers, along with low sheet resistance after activation (Supplementary Fig. 34).

To demonstrate the potential of LNEP for large-area wearable devices, we designed various sensors for on-skin detection, including a mechanical sensor array for detecting applied pressure and a multifunctional sensor for monitoring internal body signals. To capture body signals effectively, the electrodes must be conformally attached to the human body. Therefore, we fabricated LNEP in SEBS, which exhibits a skin-like modulus, stretchability, and robustness, enabling conformal attachment to the skin for reliable signal detection.

**Fig. 4:**
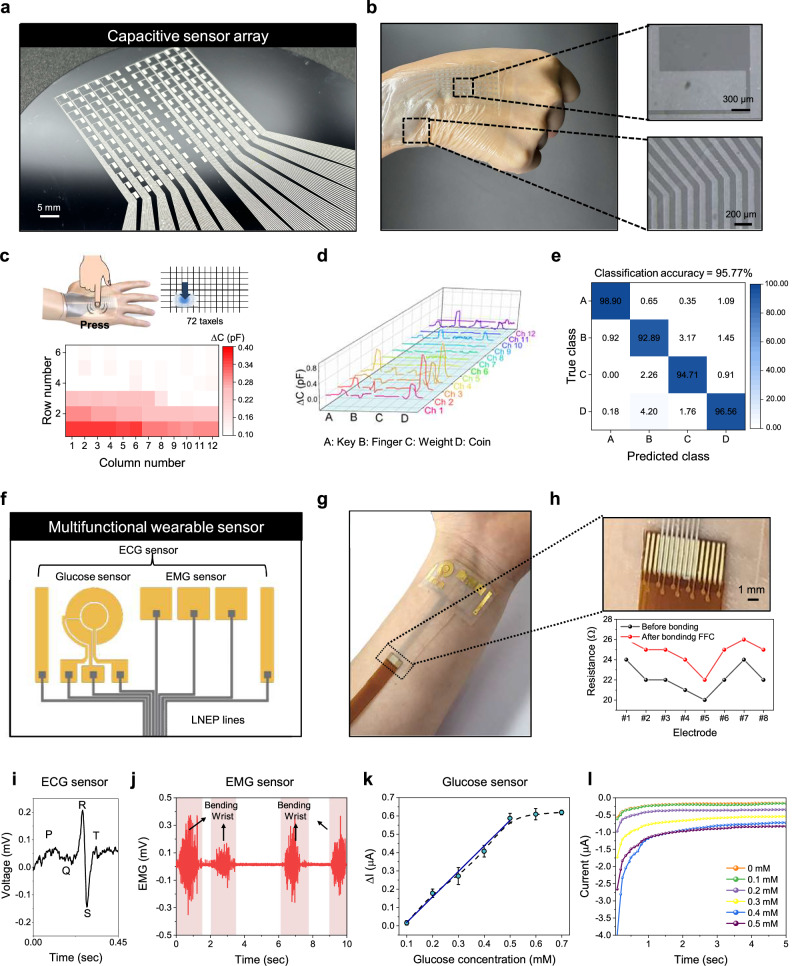
Large-area LNEP for a wearable sensor. **a** Photograph of wafer-scale patterned LMP film used for a wearable capacitive sensor array. **b** Photograph of a 72-channel (6 × 12) wearable capacitive sensor array based on LNEP. **c** Visualized taxel images from the capacitive sensor array when pressed. **d** Acquired capacitive signals from the sensor array when four different objects (key, finger, weight, coin) are pressed onto the sensor array. **e** Classification accuracy of object recognition using the capacitive sensor array through machine-learning based on a convolutional neural network (CNN). **f** Schematic illustration of the multifunctional wearable sensor. **g** Photograph of an on-skin multifunctional wearable sensor. **h** Photograph of LNEP lines bonded to a flexible flat cable (top) and the resistance measured before and after bonding with the flexible flat cable (bottom). **i** Real-time ECG signal collected with the LNEP wearable multifunctional sensor. **j** Real-time EMG signal collected with the LNEP wearable multifunctional sensor. **k** Current change of a glucose sensor according to the concentration of glucose. **l** Chronoamperometric responses of a glucose sensor according to the concentration of glucose.

To fabricate a large-area mechanical sensor array, we first patterned electrodes and interconnects with LMPs on a 4-inch Si wafer (Fig. 4a). Thereafter, the LMP patterns were transferred to the SEBS substrate. Leveraging the mechanical properties of SEBS, our LNEP-based mechanical sensor array was conformally adhered to a human hand and further secured with medical tape (Fig. 4b). When loading pressure was applied to the 6×12 taxel array, the capacitance of each sensor increased in proportion to the applied pressure (Fig. 4c). Using this taxel array, real-time monitoring of 12 channels was conducted, and capacitance signals were recorded for 4 different objects (key, finger, weight, coin) that were each placed on the hand 30 times (Fig. 4d). The collected data were successfully classified into four categories with accuracy of 95.77% based on a convolution neural network (Fig. 4e and Supplementary Fig. 35).

To detect human body signals, we fabricated a multifunctional wearable sensor incorporating glucose, electrocardiogram (ECG), and EMG sensors using LNEP in SEBS and Cr/Au electrodes (Fig. 4f,g and details of the fabrication process are provided in the “Methods” section). To access the multifunctional wearable sensor, LNEP lines were bonded to a flexible flat cable (FFC) using anisotropic conducting film (ACF) tape (Fig. 4h, top and Supplementary Fig. 36). After bonding the LNEP with the FFC, the resistance of the 8 electrodes remained low, indicating a stable connection with the FFC (Fig. 4h, bottom).

ECG signals were monitored using the multifunctional wearable sensor connected through the FFC cable. High-fidelity ECG signals were obtained, with clear PQRST waveforms successfully recorded (Fig. 4i and Supplementary Fig. 37). Similarly, we successfully observed EMG signals by attaching the sensor to the forearm during repeated wrist-bending movements (Fig. 4j).

The electrochemical glucose sensor was fabricated by functionalizing the working electrode (WE) with the enzyme glucose oxidase (details of the glucose sensor fabrication process are provided in the Methods). Through the redox enzyme-immobilized WE, the current level increased due to hydrogen peroxide generated from the enzymatic reaction, corresponding to the glucose concentration (Fig. 4k). The response of the glucose sensor was evaluated over a glucose concentration range of 0.1–0.7 mM, with a linear response observed up to 0.5 mM. The chronoamperometric (CA) response was also measured within the same glucose concentration range (Fig. 4l). These results verify the potential of LNEP as a stretchable interconnect for future wearable applications.

To achieve universal applicability of LNEP as a stretchable conductor in stretchable electronics, robust electrical and mechanical connections with rigid electronics under deformation are essential. Previously, rigid chips were integrated with interconnectors using stretchable solders^49,50^ or by sealing them with elastomers^51,52^. However, these methods are challenging to apply to high-resolution devices, and it is difficult to utilize chip bonder equipment on soft elastomer substrates. Also, for the sealing method, an additional process is required to create contact pads for wiring.

**Fig. 5:**
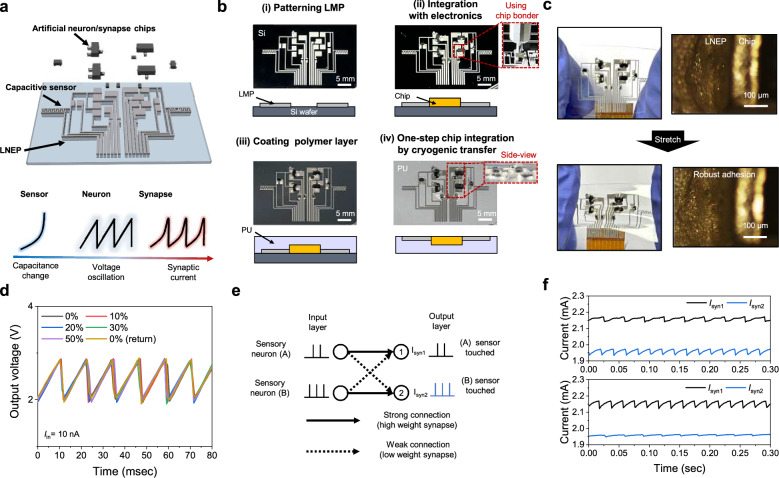
Stretchable neuromorphic circuit utilizing LNEP as interconnects integrated with rigid electronic chips. **a** Schematic illustrations of the fabricated neuromorphic circuit (top) and its working principle (bottom). **b** Photographs of the one-step chip integration process. **c** Photographs of the LNEP-based stretchable neuromorphic circuit (left) and optical microscope images of the chip interface (right), shown before stretching (top) and after stretching (bottom). **d** Iterative spiking characteristics of the neuron device with LNEP interconnects under 0% to 50% strain. **e** Spiking neural network (SNN) for touch classification. The input layer represents two artificial sensory neurons, while each output layer corresponds to the sensor being touched. **f** Synaptic currents were measured at both output layers (*I*_syn1_ and *I*_syn2_). When the frequency of *I*_syn1_ (*f*_I1_) exceeded that of *I*_syn2 (_*f*_I2)_, the circuit identified the (B) sensor as being touched. Conversely, when *f*_I1_ was lower than *f*_I2_, the circuit identified the (A) sensor as being touched.

Our cryogenic transfer enables robust adhesion with rigid chips using chip bonder equipment in a one-step transfer process. Using this method, we could fabricate a stretchable touch-sensory neuromorphic circuit composed of 2 neurons and 4 synapses (Fig. 5a, top). A bipolar junction transistor (BJT) was used for an artificial neuron that generated spiking signals, while a metal-oxide-semiconductor field-effect transistor (MOSFET) connected to a resistor in series (abbreviated as 1T1R) was used as a synapse to control the synaptic weights (Supplementary Fig. 38). When the sensor of the circuit was touched, the capacitance of the sensor changed, inducing a change in the frequency of the output voltage (*V*_out_) of the neuron device, which subsequently generated spike-shaped synaptic currents (*I*_syn_) (Fig. 5a, bottom).

To fabricate a neuromorphic circuit, we first patterned the LMP lines and applied solder paste to the areas designated for chip integration (Fig. 5b(i)). And then we integrated the rigid chips through thermocompression at 180 °C for 30 s using a chip bonder (Fig. 5b(ii)) and Supplementary Fig. 39. Afterward, we coated polyurethane (PU) and transferred the all LMP lines and chips simultaneously into the polymer matrix in a one-step using the cryogenic transfer (Fig. 5b(iii–iv)). PU was used as the substrate due to its high toughness and robustness^45^. The transferred LNEP exhibited robust adhesion with the chips under strain and showed only an 87% increase in resistance after being stretched to 100%, comparable to the 90% increase observed in LNEP alone at 100% strain (Fig. 5c and Supplementary Fig. 40).

This fabrication process ensures the reliable integration of LNEP and rigid chips to function as the stretchable neuromorphic circuit. The BJT-based artificial neuron device in the neuromorphic circuit utilizes the single transistor latch (STL) phenomenon to mimic the integrate-and-fire (IF) operation of a biological neuron (Supplementary Fig. 41a, b). To operate the touch-sensory neuron module, a constant operating current (*I*_in_) was applied to the collector (C) of the BJT-based neuron, generating output voltage (*V*_out_) with an oscillation frequency (*f*_V_), which was also measured at the same collector. Here, *f*_V_ can be modeled as ${f}_{{{\rm{v}}}}=\frac{{I}_{{in}}}{{C}_{{sen}}({V}_{{top}}-{V}_{{bot}})}$, where *C*_sen_ is the capacitance of the sensor, and *V*_top_ and *V*_bot_ are the top and bottom of *V*_out_, respectively (Supplementary Note 1 and Supplementary Fig. 41c). Due to its robust adhesion, the artificial neuron with LNEP interconnects maintained consistent spiking characteristics even under strains ranging from 0% to 50%, demonstrating the resilience to mechanical deformation (Fig. 5d and Supplementary Fig. 41d).

Using the stretchable neuromorphic circuit, we designed a SNN to classify the touched location (Fig. 5e). When a capacitive touch sensor connected in parallel with the BJT-based neuron is stimulated, an oscillating *V*_out_ is produced from the BJT-based neuron, and this spiking voltage signal is applied to the 1T1R synapses. Consequently, a resultant synaptic current (*I*_syn_) with an oscillation frequency (*f*_I_) is generated. In the simplified 2 × 4 SNN configuration (2 neurons with 4 synapses), one artificial neuron connected to sensor (A) had a strong connection to output layer 1 and a weak connection to output layer 2. In contrast, the artificial neuron connected to sensor (B) had a strong connection to output layer 2 and a weak connection to output layer 1. The intensity of these connections was controlled by the resistance in the 1T1R synapse (Supplementary Fig. 42).

Finally, using the assembled stretchable neuromorphic circuit, we classified the predominant touched location. When the area near the sensor (A) was touched, *f*_I1_ of *I*_syn1_ decreased due to the increase in *C*_sen(A)_. In contrast, when the area near the sensor (B) was touched, *f*_I2_ of *I*_syn2_ decreased due to the increase in *C*_sen(B)_. Consequently, the touched location is classified by the difference in the *f*_I_ values of the synaptic currents.

**Fig. 6:**
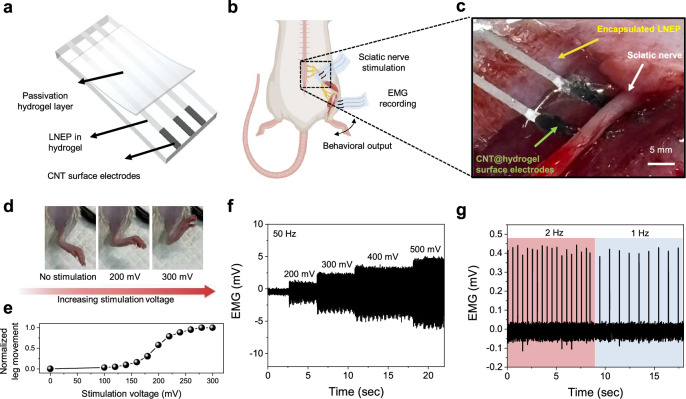
In vivo applications using LNEP. **a** Schematic of the LNEP-based in vivo device for EMG recording and sciatic nerve electrical stimulation. Multi-wall carbon nanotubes were used as the surface electrode. **b** Schematic of sciatic nerve electrical stimulation with simultaneous EMG recording. **c** Photograph of the LNEP-based device attached to the sciatic nerve. **d** Photograph of leg movement depending on the stimulation voltage. **e** Normalized leg movement as a function of the stimulation voltage. **f** Recorded EMG signal during nerve stimulation at 50 Hz with an increase in the voltage level. **g** Recorded EMG signal during nerve stimulation at different frequencies of 2 Hz and 1 Hz. **b** was created in BioRender. Rachim, V. (2025) https://BioRender.com/xd13cby.

The universality of LNEP also enables its application to biocompatible hydrogels, which are in high demand for implantable bioelectronics^53^. In order assess whether LNEP can be utilized in in vivo environments, we first deposited a passivation hydrogel layer and surface electrodes onto the LNEP in hydrogel (Fig. 6a). The fabricated LNEP-based device was then attached to the sciatic nerve and the tibialis anterior muscle of a rat to stimulate the hindlimb and record EMG signals, respectively (Fig. 6b, c). As the applied voltage to the nerve was increased from 0 mV to 300 mV, the movement of the hindlimb increased (Fig. 6d, e and Supplementary Fig. 43). Furthermore, the recorded EMG signals increased in proportion to the stimulation voltage, demonstrating successful stimulation of the leg muscle (Fig. 6f). Also, the EMG signals were successfully recorded during constant-frequency stimulation input (Fig. 6g). Finally, the in vitro biocompatibility and long-term operation of the LNEP-based bioelectronics were demonstrated to ensure the reliability of this device when implanted (Supplementary Figs. 44, 45).

## Result and discussion

We have developed a universal high-resolution method for stretchable electronics by integrating liquid metal particle networks (LMPs) into diverse polymer substrates. Using etching-based photolithography and a cryogenic transfer process, this approach addresses all key attributes needed in commercially viable liquid metal patterning: large-area scalability, uniformity, high-yield, and substrate versatility. The resulting liquid metal-embedded polymer (LNEP) exhibits excellent electrical conductivity (~1.71 × 10⁶ S/m), low gauge factor, and cyclic stability. This method supports a wide range of applications by leveraging the mechanical and functional properties of different polymers. LNEP was employed to create wearable capacitive sensor arrays with precise touch classification capabilities. Using a one-step transfer process, rigid functional chips were integrated with LNEP, enabling the fabrication of stretchable neuromorphic circuits that maintained stable performance under strain. Additionally, the cryogenic transfer process facilitated the integration of LNEP into biocompatible hydrogels, demonstrating potential for in vivo applications such as nerve stimulation and EMG recording. While further optimization is needed for long-term stability and broader material compatibility, this method provides a unique solution for the commercialization of LM-based stretchable electronics, as it will offer broadly applicable robust electronic platforms for various demands in the future.

## Methods

### Materials

Unless otherwise specified, all the chemicals used in this work were purchased from Sigma-Aldrich and utilized without additional purification. Multi-walled CNT was purchased from Applied carbon nano, Gallium, Eutectic gallium indium (EGaIn), and Galinstan were purchased from Changsha Ruichi Nonferrous Metals. N,N-Dimethylacetamide (99%), hydrochloric acid (37%), hydrogen peroxide (35%) hexamethyldisilazine (99.9%), toluene (99.5%) octadecyltrichlorosilane (90%), acetic acid (99%), ethyl acetate (99.8%), bovine serum albumin, reduced graphene oxide-tetraethylene pentaamine, 70% glutaraldehyde, PBS, MES buffer, 1-ethyl-3-(3-dimethylaminopropyl) carbodiimide hydrochloride (EDC), N-hydroxysuccinimide (NHS), glucose oxidase from *Aspergillus niger*, ethanolamine, Tween 20, and D-(+)-Glucose were purchased from Sigma-Aldrich. Photopatterning of LMP was conducted using the positive photoresists AZ GXR-601 and AZ 10XT (AZ Electronic Materials), along with AZ300MIF developer (AZ Electronic Materials). For the elastic polymer matrix, we purchased polydimethylsiloxane sylgard 184 (PDMS) from Dow Corning, pluronic^®^ F-127, acrylamide, sodium chloride, *N,N’*-Methylenebis(acrylamide), α-Ketoglutaric acid, agarose, dichloromethane (DCM), methacryloyl chloride, triethylamide (TEA), 2-hydroxy-4’-(2-hydroxyethoxy)−2-methylpropiophenone (Irgacure 2959), polyurethane (PU), and polyamic acid (PAA) solution from Sigma-Aldrich, and styrene ethylene/butylene styrene (TUFTEC H1221, SEBS) from Asahi Kasei. PDMS was mixed in a 10 (base):1 (curing agent) weight ratio. SEBS was diluted to a 10% concentration with toluene solvent. PU was diluted to a 20% concentration with N,N-Dimethylacetamide solvent. The part A and the part B of the Dragon skin 10 NV were mixed in a 1:1 weight ratio with a planetary mixer (Thinky AR-100) to perform a mixing time of 2 min and a deformation time of 30 s. Polyimide film was fabricated by imidization of PAA. Tegaderm (3 M) medical tape was used to attach the wearable sensor to the human body. To bond LNEP to FFC, ACF tape was purchased from 3 M.

### Preparation of PF127DMA-AAm hydrogel^54^

Firstly, Pluronic F127 dimethacrylate (PF127DMA) was synthesized according to a protocol. 2.1 g of PF127DMA was added to 4 mL of DI water with magnetic stirring at a low temperature for 8 h. After PF127DMA was homogeneously dissolved, 1.5 g of acrylamide, 0.009 g of MBA, and 0.054 g of I2959 were added, and thoroughly mixed. The precursor was then cured using an area-type UV curing system (LGA10100F, Liim Tech) for 60 s.

### Preparation of LMP ink

LMP ink was prepared by mixing 2 g of EGaIn, 0.2 mL of acetic acid, and 4 mL of ethyl acetate in a 20 mL vial. The mixture was subjected to a tip sonication (VC-505, Sonics & Materials, 12.7 mm solid tip) at 60% amplitude for a duration of 5 min.

### Patterning of LMP film

The following steps were carried out for the patterning of the LMP film on a Si wafer. (1) A spin-coated LMP film on the Si wafer was prepared. (2) Bis(trimethylsilyl)amine (HMDS) from AZ electronic Materials was coated using a vapor deposition process for 20 min at 120 °C to enhance the adhesion of photoresist. (3) Positive photoresist was spin-coated on the film and baked at 90 °C for 3 min. The spin-coating, baking, and exposure conditions were adjusted based on the product and thickness of the photoresist. (4) The photoresist was exposed at a dose of 100 W using the i-line (365 nm) with a mask aligner (Midas, MDA-8000B), followed by a post-exposure bake at 110 °C for 3 min. (5) After cooling to room temperature, the exposed film was developed with the AZ300MIF developer for 30 s, then rinsed with deionized water and dried with an N_2_ gun. (6) A hard bake was performed at 180 °C for 20 min to prevent film flaking during the wet-etch process. (7) The wet-etch process was conducted for 10 s to remove unprotected LMP areas with the 1:1 mixture of the hydrochloric acid and hydrogen peroxide. After the etch process, the LMP film was rinsed several times to clean up the remnant etchant with deionized water. (8) To remove the photoresist, the patterned LMP film was then immersed in an acetone bath for 3 min, and a min spray to remove any remaining photoresist. Afterwards, it was rinsed with ethanol and deionized water for several times, followed by drying using the N_2_ gun.

### Fabrication of LMP/SEBS composite

For the control group, LMP/SEBS composite was used. Initially, LMP ink was centrifuged with an AR-100 mixer for 3 min in a defoaming mode. Then, the solvent was gently decanted. Next, the SEBS polymer solution was prepared by dissolving SEBS in toluene at a weight ratio of 1:1 and stirring it for 24 h at 80 °C. Then, 0.2 g of the SEBS solution was added to the LMPs (LMPs at 75 vol%) and mixed using the AR-100 for 3 min in a mixing mode. The mixture was poured onto a Si substrate, and heating 1 h in 80 °C. After the drying process, the film was detached from the substrate.

### Material characterizations

The optical images of patterned LMP structures were obtained by a scanning electron microscope (Hitatch, SU5000). In-situ tensile test of film was conducted using a scanning electron microscope (Thermo Scientific, Quattro S) equipped with a tensile stage (Microtest, 2kN module). Profiles of elemental mapping were obtained using the same equipment. Cryo-FIB equipment (Thermo Scientific, Aquilos) was used to obtain SEM images under different temperatures. Contact angle analyzer (SEO Pheonix, Pheonix 300 touch) was used to determine the wettability of the LMP ink. The surface analysis after transferring the LMPs was conducted using an XPS (Axis-Supra, Kratos). To analyze the crystal structure of gallium oxide at different temperatures, temperature X-ray diffractometer (RIGAKU, SmartLab) was used. The surface profile was analyzed through a 3D profiler (Bruker, ContourGT).

### Electrical characterization

Electrical conductivity was measured for LNEP line (bar-pattern with 1 mm **×** 1 mm pad) using a 4-point probe (MSTech, M4P205), and series measure meter (Keithley, 2401). The thickness of LMP films was measured from SEM (Hitachi, SU5000) images. To measure the resistance changes under strain, a force gauge (Mark-10, M5), a motorized stand (Mark-10), and an LCR meter (HP Agilent Keysight, 4284 A) were used. To form the VIAs, a CO_2_ desktop laser machine (OMTech, 40 W) was used with a power of 12.0% and a writing speed of 20 mm/s.

### Measurement of the wearable capacitive sensor array

Capacitive sensor measurements were performed using the commercial FDC2214EVM (Texas Instruments) evaluation module. The module was configured to operate at a sampling rate of 200 Hz with a resolution of 28 bits. The system was calibrated prior to data acquisition using a reference capacitance of 10 pF to ensure accuracy.

### Preparation of the multifunctional wearable sensor

The multifunctional wearable sensor consists of ECG sensor, EMG sensor, and electrochemical biosensor. For the interconnects of the abovementioned sensors, LMP lines were patterned and transferred to the SEBS polymer using the previously described methods. Afterward, sensing electrodes were fabricated by depositing a metallic source (Cr/Au = 10 nm/100 nm) onto the LMP-patterned SEBS substrate using an e-beam evaporator (SNTEK, Co., Ltd.).

### Preparation of the electrochemical biosensor

The electrochemical biosensor consists of a working electrode, a reference electrode, and a counter electrode. The working electrode was drop-casted with a solution containing bovine serum albumin (BSA) (Sigma Aldrich, USA, no. A7906), reduced graphene oxide-tetraethylene pentaamine (prGOx) (Sigma Aldrich, USA, 806579), and 70% glutaraldehyde (Sigma Aldrich, USA, no. G7776) in PBS (Sigma-Aldrich, USA, no. D8537) for a transducing layer. This coating method has been fully optimized previously^55^. The biosensor was first treated with a solution containing 50 mM MES buffer (Sigma-Aldrich, USA, no. M1317), 400 mM 1-ethyl-3-(3-dimethylaminopropyl) carbodiimide hydrochloride (EDC) (Sigma Aldrich, USA, no. E7750), and 200 mM N-hydroxysuccinimide (NHS) (Sigma Aldrich, USA, no. 130672) for 30 min. The biosensor was washed three times with PBS. After that, the working electrode was spotted with 40 mg mL^−1^ of glucose oxidase from Aspergillus niger (Sigma Aldrich, USA, no. 130672) for 3 h at 4 °C. Subsequently, the working electrode was washed three times with PBS. Any remaining unreacted functional groups were blocked with 1 M ethanolamine (Sigma-Aldrich, USA, no. 398136) in PBS for 30 min, followed by a washing step with PBS. The working electrode was then incubated with a blocking buffer containing of 1% BSA and 0.05% Tween 20 (Sigma Aldrich, USA, P1379) in PBS for 1 h at 4 °C. Finally, the working electrode was washed with PBS containing 0.05% Tween 20 (PBST).

### Amperometric glucose detection

The as-prepared biosensor was used to detect D-(+)-Glucose (Sigma Aldrich, USA, G7528) against the immobilized glucose oxidase. Chronoamperometry (CA) measurements were conducted in PBS, and the working electrode was polarized at a constant potential level of −0.3 V for 5 s. For these measurements, 200 μL of the solution was continuously incubated by adding serially diluted glucose solution. The response for each CA measurement was recorded after reaching a steady state.

### Measurement of the multifunctional wearable sensor

The real-time monitoring of ECG, and EMG signals was conducted with commercial wireless equipment (Bitalino). For measuring the ECG, a commercial gel electrode (3 M) was used as the reference electrode.

### Fabrication of the neuromorphic circuit

The neuromorphic circuit is composed of 2 capacitive touch sensors, 2 BJTs (BFP840FESDH6327XTSA1, Infineon Technologies), 4 MOSFETs (BSS138LT1G, Onsemi), and 4 resistors (1608 size). The LMP lines were patterned onto a Si substrate to facilitate interconnection within the neuromorphic circuit. Subsequently, chips were aligned and bonded to the wafer using a chip bonder (Finetech, fineplacer pico ma/rs). PU was then spin-coated onto the wafer. Finally, both the LMP interconnects and the chips were simultaneously transferred to the PU substrate using the cryogenic transfer.

### Electrical characterization of neuromorphic circuit

Electrical characterization of the neuromorphic circuits was conducted using a B1500A parameter analyzer (Keysight). The output synapse current was measured using a 428 current amplifier (Keithley) and a TDS 744A oscilloscope (Tektronix).

### Computational methods of AIMD calculations

All calculations from first principles were conducted using the Vienna Ab initio Simulation Package (VASP) with the revised Perdew–Burke–Ernzerhof for solids (PBEsol) form of the Generalized Gradient Approximation (GGA) functional. To account for dispersion interactions accurately, we utilized the DFT-D3 method with Becke–Johnson damping. Initial crystallographic structures, including α-Ga₂O₃^56^, Ga(III)^57^ in its liquid phase, β-Ga^58^, and Si^59^, were prepared with appropriate facet orientations and obtained from verified structural databases. These initial structures were then optimized to create slab models using the PBEsol functional with a kinetic energy cutoff of 600 eV. For the optimization process, convergence criteria were set such that atomic forces were minimized below 0.01 eV Å⁻¹, and the total energy for each structure reached convergence within 10⁻^6^ eV during the self-consistent iterations.

Once the slabs were fully optimized, heterostructures were created by stacking the slabs with varying distances between the layers. SCF energy calculations were performed iteratively by adjusting the interlayer distance to find the configuration with the lowest energy, which indicated the most stable separation between the slabs in each heterostructure.

To explore thermal effects, we carried out AIMD simulations on the constructed heterostructures and each individual structure. Each AIMD simulation ran for 3000 steps with a time step of 1 fs at two temperatures, 77 K and 298 K, to study temperature-dependent interactions. Average energies were obtained from the final 1000 steps of each simulation to ensure equilibrium was reached at each temperature. From these simulations, we calculated binding energies at both temperatures to evaluate thermal stability and interfacial interaction strengths.

### Fabrication of the in vivo device

Hydrogel was synthesized prior to the fabrication of the device. To synthesize the hydrogel, 4.4 g of acryl amide, 0.004 g of N,N’-Methylenebis(acrylamide), and 0.01 g of α-Ketoglutaric acid were added to 10 mL of DI water with magnetic stirring at a 92 °C for 5 h. After that, 0.7 g of agarose was added and homogeneously dissolved. The precursor was then cured using an area-type UV curing system for 60 s.

For the surface electrode, CNT@hydrogel was fabricated by mixing the hydrogel precursor with 10 wt% MWCNT using a planetary mixer for 1 min. The LNEP in hydrogel was prepared using the aforementioned cryogenic transfer with patterned LMP lines. For the surface electrodes, CNT@hydrogel was applied onto the LNEP lines and cured under the same conditions, after which the exposed LNEP lines were encapsulated with hydrogel.

### In vivo sciatic nerve stimulation

Male Sprague Dawley rats (8 weeks old, 270–300 g) purchased from Orient Bio Inc. were used for the in vivo experiments. After the application of anesthesia, the hair of rat was removed on the hindleg skin, and the sciatic nerve was exposed by separating the gluteus maximus and bicep femoris muscles. LNEP based electrodes were placed under the sciatic nerve with two electrodes connected for electrical stimulation. An isolated pulse stimulator (33220A, Agilent) was used to apply electrical stimulation. The EMG signals were recorded simultaneously by MP160, BIOPAC. All procedures were approved by the Pohang University of Science and Technology Institutional Animal Care and Use Committee (POSTECH IACUC, POSTECH-2024-0026).

### In vitro biocompatibility test^60^

In vitro biocompatibility was evaluated by incubation of LNEP-based in vivo device in cell media. Normal human dermal fibroblasts (NHDFs) were grown in Dulbecco’s Modified Eagle’s Medium (DMEM, Gibco) supplemented with 10% (v/v) heat-inactivated fetal bovine serum (FBS) and 1% (v/v) penicillin/streptomycin (P/S, Gibco). To evaluate the biocompatibility, the material was immersed in culture medium for 1 day. 2 × 10⁵ cells were plated onto a 35 mm culture dish (SPL) and incubated for 24 h. After washing the culture plates with Dulbecco’s phosphate-buffered saline (DPBS), and stained with a LIVE/DEAD viability/cytotoxicity kit for mammalian cells (Thermo Fisher Scientific). Each sample was incubated with DPBS (1 mL) containing ethidium homodimer-1 (4 µm) solution and calcein-AM (2 µm) for 10 min at 37 °C, followed by rinsing all samples with DPBS. The numbers of viable and non-viable cells were analyzed using Image J software.

### Experiments on human subjects

Two healthy male participants (aged 30), who are also authors of this study, were recruited to evaluate the wearable performance. Sex and gender were determined based on self-report. No compensation was provided. All experiments, including demonstrations on human skin, were performed under approval from the Institutional Review Board at KAIST (protocol number: KH2024-110), and informed consent was obtained from the volunteer subjects. Sex and gender were not considered in the study design. The primary objective was to validate the feasibility of our device regarding human motion or pressure, rather than to investigate biological variations based on sex or gender.

### Statistics and reproducibility

No statistical method was used to predetermine sample size. The experiments corresponding to Figs. 1a, 2a, b, e, 3g, and 5c were repeated independently at least five times with similar results.

### Reporting summary

Further information on research design is available in the Nature Portfolio Reporting Summary linked to this article.

## Supplementary information

Supplementary Information

Description of Additional Supplementary Information

Supplementary Video 1

Reporting Summary

Transparent Peer Review file

## Source data

Source data

## Data Availability

The authors declare that all data supporting the findings of this study are available within the paper, Supplementary Information, and Source Data file. All other data are available from the corresponding authors upon request. Source data are provided with this paper.
