# Supplementary material for: Universal cryogenic transfer of liquid metal particles in polymers for wafer-scale stretchable integrated electronics
Source: Nat Commun. 2026 Feb 26;17:3248. doi: 10.1038/s41467-026-70101-2 (PMC13061968; doi:10.1038/s41467-026-70101-2)
Supplement: Supplementary file 1 — Supplementary Information [file 41467_2026_70101_MOESM1_ESM.pdf]

Table S1 | Comparison of liquid metal patterning methods between this work and previous works[1-21].

Patterning strategy	Approach	Phase	Resolution	Elastomer substrate	Scalability	Thickness control	Thickness uniformity	Alloying issue	Leakage of liquid metal
Lithography-enabled process	**Etch & transfer (This work)**	**Liquid metal particle**	**5 μm**	**Universal**	**Wafer-scale**	**Available**	**Uniform**	**Free**	**No**
	e-beam lithography & Stamping[1]	Bulk liquid metal	180 nm	PDMS	Limited (Low throughput EBL)	Available	Uniform	Free	Yes (encapsulation layer necessary)
	Metal lift-off & selective wetting[2-4]	Bulk liquid metal	2 μm	Universal except hydrogel	Wafer-scale	Limited (Thickness proportional to line width)	Convex surface	Exist (Solid-phase intermetallic compound)	Yes (encapsulation layer necessary)
	Lift-off[5]	Bulk liquid metal	20 μm	PDMS	Wafer-scale	Limited (Thickness proportional to line width)	Convex surface	Free	Yes (encapsulation layer necessary)
	Lift-off[6]	Liquid metal particle	20 μm	PDMS	Wafer-scale	Available	Uniform	Free	No
	Microchannel & transfer[7-8]	Bulk liquid metal	2 μm	PDMS	Wafer-scale	Available	Uniform	Free	Yes (encapsulation layer necessary)
Injection	Microchannel & vacuum filling[9-10]	Bulk liquid metal	5~70 μm	PDMS	Wafer-scale	Limited (Require high channel thickness)	Uniform	Free	Yes (encapsulation layer necessary)
Printing	Direct printing & transfer[11]	Bulk liquid metal	50 μm	PDMS	Wafer-scale	Limited	Non-uniform	Free	Yes (encapsulation layer necessary)
	Direct printing[12]	Liquid metal	30~83 μm	PET, PDMS	Limited (Slow printing speed, 0.2 mm/s)	Limited (Thickness proportional to line width)	Convex surface	Free	Yes (encapsulation layer necessary)
	Inkjet printing[13]	Liquid metal particle	50 μm	Hydrophilic polymers	Wafer-scale	Available	Uniform	Free	No
	Screen printing[14]	Liquid metal particle	200 μm	Various polymers	Wafer-scale	Available	Uniform	Free	No
	Metal stencil coating & selective wetting[15-16]	Bulk liquid metal	5 μm	Universal	Wafer-scale	Limited (Thickness proportional to line width)	Convex surface	Exist (Solid-phase intermetallic compound)	Yes (encapsulation layer necessary)
	Microtransfer printing[17]	Liquid metal particle	5 μm	Adhesive substrates (VHB, PDMS, O^2-treated glass and Si)	Large-scale	Available	Uniform	Free	Yes (encapsulation layer necessary)
	Electrohydrodynamic printing[18]	Bulk liquid metal	19.28 μm	PDMS	Limited (Slow printing speed, 0.4-2.0 mm/s)	Available	Uniform	Exist (Solid-phase intermetallic compound)	Yes (encapsulation layer necessary)
	Electrohydrodynamic printing[19]	Liquid metal particle	1.47 μm	PDMS, Glass	Large-scale	Available	Uniform	Free	No
	Commercial printing[20]	Liquid metal particle	80 μm (stencil printing)	Various polymers	Large-scale	Available	Method-dependent	Free	No
Laser ablation	Metal wetting & direct laser writing[21]	Bulk liquid metal	4.5 μm	Universal	Limited (Low throughput)	Available	Convex surface	Exist (Solid-phase intermetallic compound)	Yes (encapsulation layer necessary)

Table S2 | Comparison of liquid metal transfer methods between this work and previous works[2,7-8,11,22-25].

Transfer strategy	Photolithography -compatibility	Transfer yield percentage (%)	Transfer detachment energy (adhesion force)	Surface average roughness (R_a) after transfer (nm)	Elastomer substrate
**Cryogenic transfer (This work)**	**Yes**	**99 <**	**3.99 mJ (PDMS)**	**152.956**	**Universal**
Stamping[2]	Yes	95 <	-	-	PDMS
Stamping[22]	Yes	-	-	-	Ecoflex
Glue[23]	No	-	-	-	Paper
Thermal transfer[24]	No	-	-	1170 (Toner)<br>760 (Knitted fabric with transferred TPU film)	Universal
Thermal transfer[25]	No	99 <	58 mN (PDMS)<br>38 mN (Paper)	-	Universal
Microchannel & transfer[7]	Yes	-	-	-	PDMS
Microchannel & transfer[8]	Yes	-	-	-	PDMS
Freezing transfer[11]	No	-	0.87 N (PDMS)	-	PDMS

Table S3 | Glass transition temperature of various polymers[26-36].

Polymer	Glass transition temperature (K)	Reference
Polydimethylsiloxane (PDMS)	150	26
Polyurethane (PU)	243	27
Polyimide (PI)	525	28
Styrene ethylene/butylene styrene (SEBS)	228.5	29
Hydrogels	273	-
Dragon skin	240	30
Polyethylene terephthalate (PET)	349.1	31
Polyvinyl chloride (PVC)	333	32
Polytetrafluoroethylene (PTFE)	170	33
Polyethylene (PE)	200	34
Polystyrene (PS)	363.6	35
Polymethylmethacrylate (PMMA)	373	36

Supplementary Note 1 | Equation for spiking frequency (*f*) of the touch-sensory artificial neuron module[37].

When an I_{in} is applied to the collector of the artificial neuron device, the output voltage at the same node (V_{out}) is modelled to the following differential equation:

$$I_{\text{in}} = C_{\text{sen}} \frac{dV_{\text{out}}}{dt}$$

, where C_{sen} represents the sensor capacitance connected in parallel to the artificial neuron.

This equation can be rewritten as

$$dt = \frac{C_{\text{sen}}}{I_{\text{in}}} dV_{\text{out}}$$

Integrating the equation over one spiking cycle, we can get the equation as

$$\int_0^{\frac{1}{f}} dt = \int_{V_{\text{bot}}}^{V_{\text{top}}} \frac{C_{sen}}{I_{in}} dV_{\text{out}} = C_{\text{sen}}(V_{top} - V_{bot})$$

From the above equation, the spiking frequency of voltage oscillation (f_{v}) can be written as

$$f_{\text{v}} = \frac{I_{in}}{C_{\text{sen}}(V_{top} - V_{bot})}$$

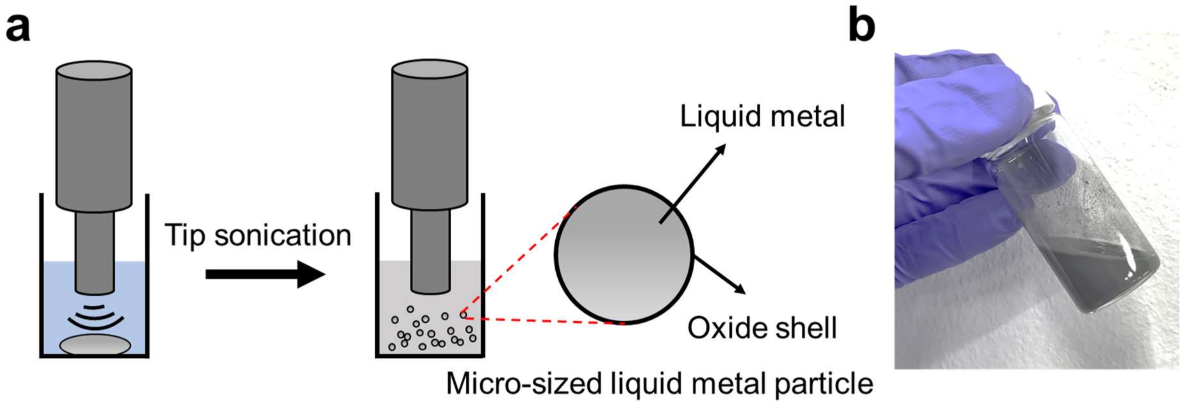

Supplementary Fig. 1 | Fabrication process of LMP ink. a, Schematic of the fabrication process. **b,** Photograph of the fabricated LMP ink. LMP ink was fabricated by LM in a mixture of ethyl acetate and acetic acid. The sonication process was done by applying mechanical energy for 5 minutes using a 12.7 mm solid tip. The spherical micro-sized LMP particles produced through tip sonication are surrounded by a thin oxide shell.

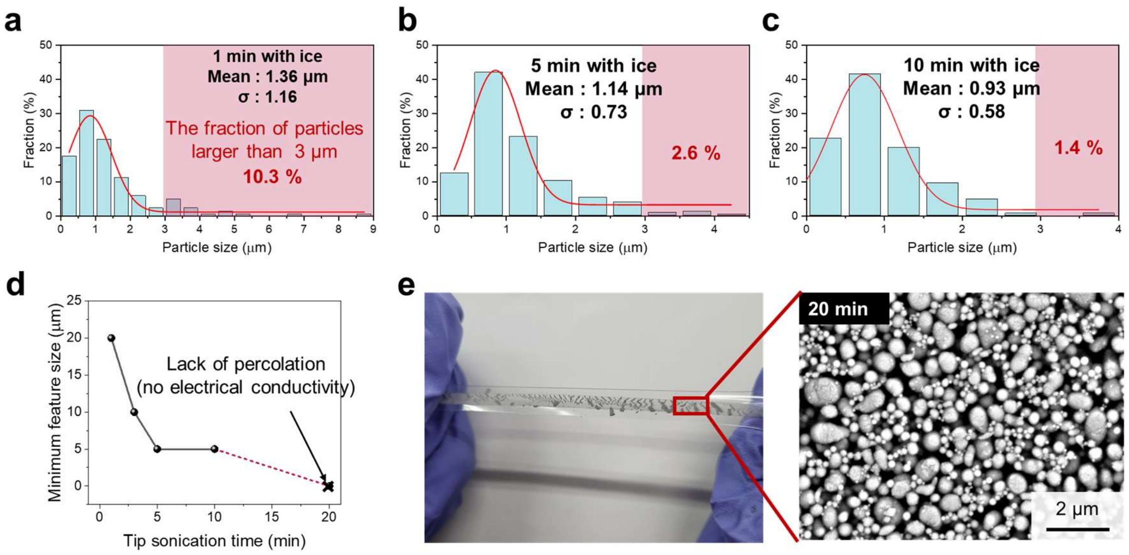

Supplementary Fig. 2 | Measured particle diameter of LMPs. a-c, Fractional distribution of LMP sizes controlled by tip sonication for 1 min (**a**), 5 min (**b**), and 10 min (**c**). **d,** Minimum feature sizes of LMPs according to tip sonication time. **e,** Optical photograph showing crack formation and mechanical failure of stretched LMP lines coated on a polymer with 20 min of tip sonicaton (left) and magnified SEM image (right).

For a tip sonication time of 1 min with ice, the average diameter of the LMPs was 1.36 μm with a standard deviation of 1.16 μm, and 10.3% of the particles were larger than 3 μm. For 5 min, the average diameter was 1.14 μm with a standard deviation of 0.73 μm, and 2.6% of the particles were larger than 3 μm. For 10 min, the average diameter was 0.93 μm with a standard deviation of 0.58 μm, and 1.4% of the particles were larger than 3 μm. As the sonication time increased, the size of LMPs decreased. For inks with a sonication time exceeding 10 min, the minimum feature size of LMPs is reduced to less than 1 μm. As a result, the LMPs fail to sustain percolation pathways, resulting in a loss of electrical connectivity.

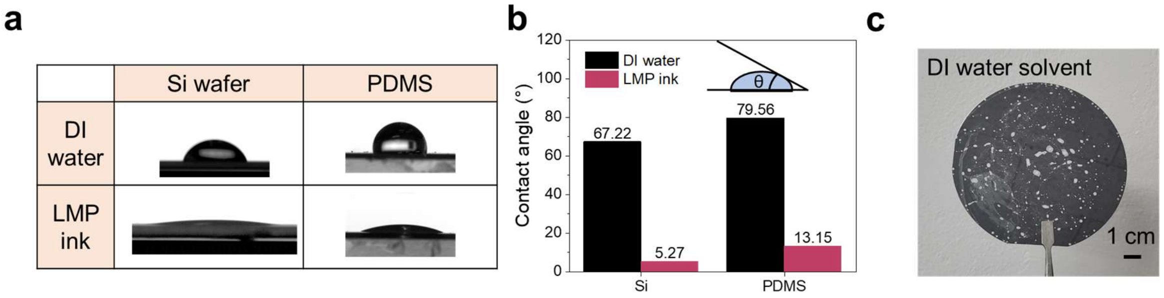

Supplementary Fig. 3 | Contact angle measurement of LMP ink. a, Photographs of LMP ink and DI water droplet on substrates. **b,** Comparison of the contact angle between LMP ink and DI water droplet. The contact angle of the LMP ink was significantly low on the silicon substrate, allowing it to spread uniformly due to its wettability. **c,** Photograph of an unevenly coated LMP film spin-coated with LMP ink using DI water as the solvent.

The contact angle of the LMP ink droplet was lower than that of the DI water droplet when deposited on Si wafer and PDMS substrates. The contact angle of LMP ink droplet was 5.27 ° on the Si wafer substrate and 13.15° on the PDMS substrate. Due to this high wettability, the LMP ink was able to be uniformly coated on both Si wafer and PDMS substrates. LMP ink using DI water as solvent was not uniformly coated on a 4-inch Si wafer due to its poor wettability with the substrate.

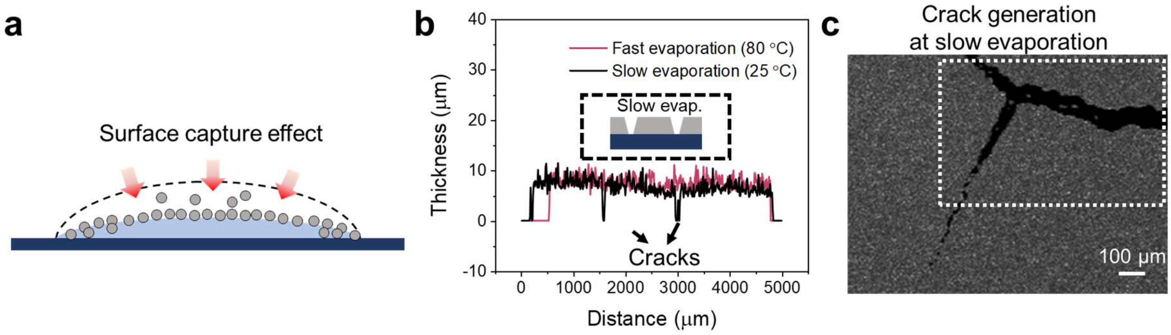

Supplementary Fig. 4 | Suppression of the coffee ring effect and crack formation by controlling evaporation kinetics. a, Schematic of the surface capture effect induced by accelerated evaporation from a volatile solvent on a heated plate. **b,** Morphology profiles of the deposited LMP film at fast evaporation (80°C) and slow evaporation (25°C). Cracks were generated during the drying process at slow evaporation due to the capillary stresses. **c,** SEM image of the generated cracks at slow evaporation.

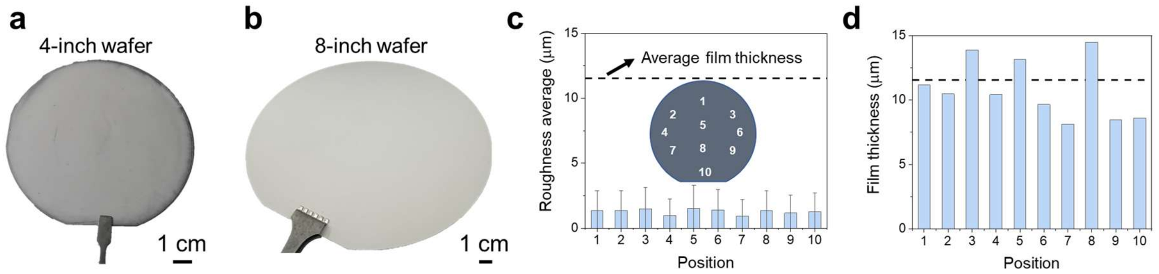

Supplementary Fig. 5 | Uniformity of spin-coated LMP films. a-b, Photograph of spin-coated LMP film with 12 mL of ethyl acetate solvent on a 4-inch Si wafer (**a**), and 4 mL of ethyl acetate solvent on an 8-inch wafer (**b**). A large solvent volume (12 mL) hinders fast evaporation, resulting in a non-uniform film. However, the optimized LMP ink with 4 mL of solvent forms a uniform film even on an 8-inch wafer. **c,** Surface roughness of the coated LMP film at various positions using 12 mL of ethyl acetate. Surface roughness of coated LMP film on a 4-inch wafer at each position with 4 mL of ethyl acetate was indicated in Fig. 2c. **d,** Film thickness of each position of the coated LMP film at various positions using 12 mL of ethyl acetate. Error bars represent mean ± standard deviation at each position.

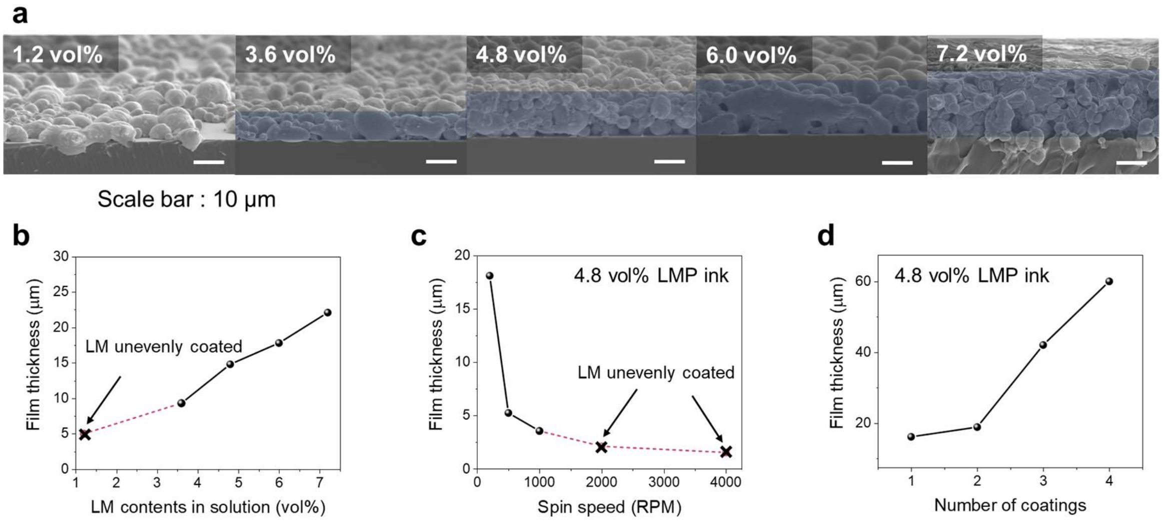

Supplementary Fig. 6 | Thickness control of spin-coated LMP films. a, Cross-sectional SEM images of spin-coated LMP films with different LM concentrations in the LMP ink. **b-d,** Average thickness of LMP films as a function of LM concentration in the solution (**b**), spin speed (**c**), and number of coatings (**d**).

The thickness of spin-coated LMP films can be controlled by the volume ratio of LM to solvent in the LMP ink. The LMP ink with 1.2 vol% LM fails to form a uniform single layer, which results in an uneven coating. As the LM content increases (3.6, 4.8, 6.0, 7.2 vol%), the thickness of the single-layer LMP film increases. During spin coating, higher spin speeds lead to a decrease in film thickness. However, at spin speeds above 2000 rpm, the LMP film becomes unevenly coated. Additionally, increasing the number of coating layers results in a thicker film. Therefore, the thickness of LMP films can be controlled by adjusting the LM concentration, spin speed, and the number of coatings.

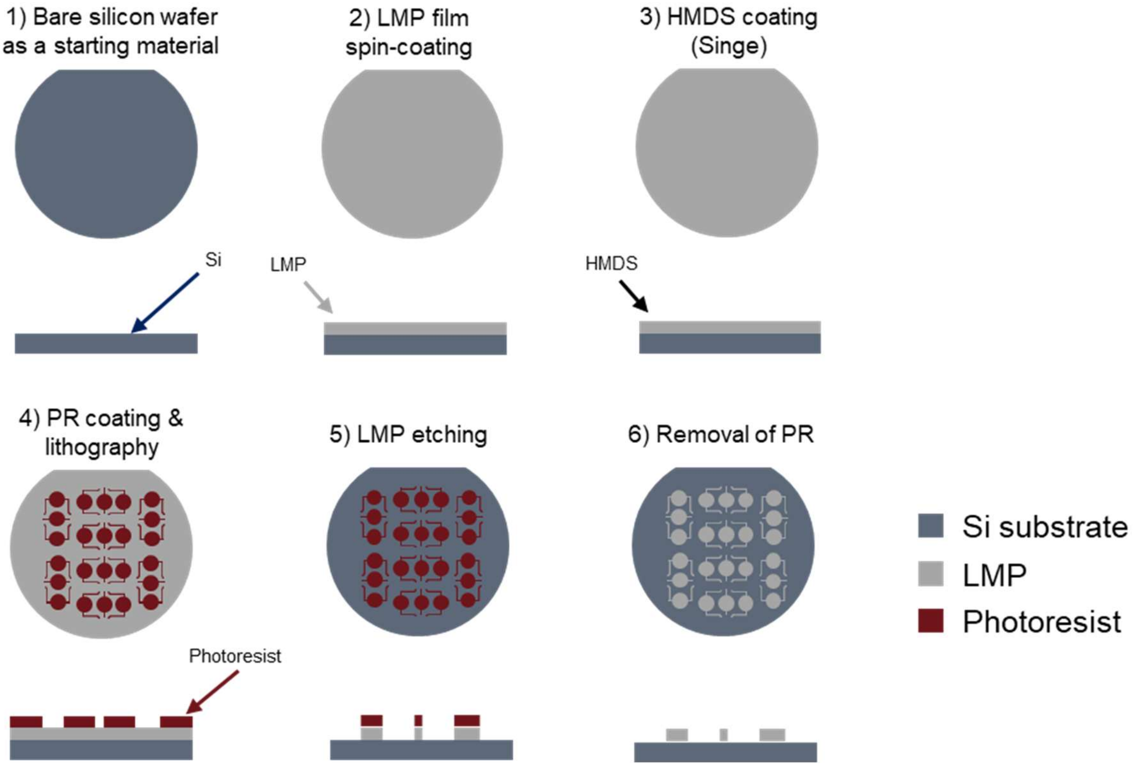

Supplementary Fig. 7 | Schematics of the patterning process of LMP using photolithography and wet etching. Detailed description of the patterning process is presented in the Method section. 1) For LMP patterning, an initial silicon wafer was prepared. 2) LMP film was spin-coated on the silicon wafer with LMP ink. 3) Hexamethyldisilazane (HMDS) was deposited onto the LMP film through chemical vapor deposition (CVD) to enhance adhesion between the photoresist (PR) and the LMP film, prior to the PR coating. Thus, the PR patterns did not delaminate from the LMP film during the subsequent PR development. 4) PR was coated on the LMP film and patterned through a top-down photolithography process. 5) The LMP film was etched with SC-2 etchant. 6) PR was removed using acetone and ethanol.

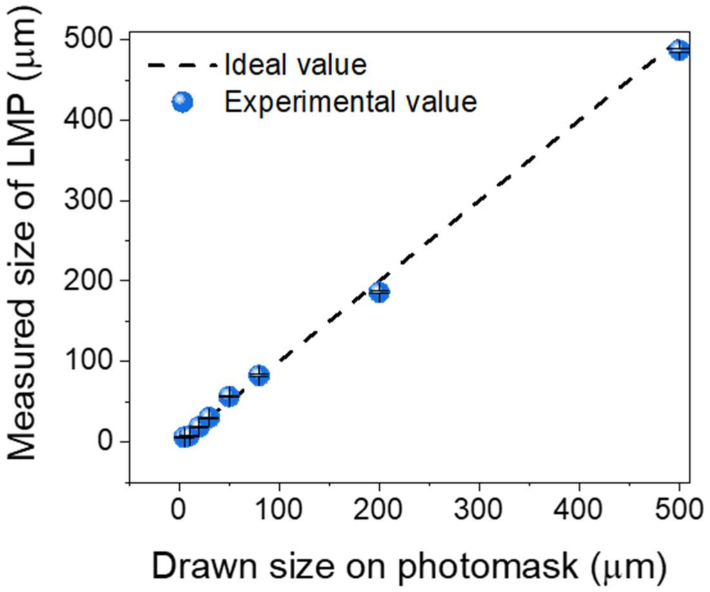

Supplementary Fig. 8 | Pattern fidelity of LMP lines. We measured the feature size of micro-patterned LMP lines at various line widths ranging from 5 μm to 500 μm. Comparison of the drawn size on the photomask (ideal value) and the actual pattern size in the LMP after etch (experimental value) shows a strong correlation. This indicates that the combinatorial approach of using top-down photolithography effectively delineates arbitrary sizes. Pattern fidelity, a critical measure of the quality and precision of the patterning process, refers to how accurately the features of a photomask or reticle are transferred onto a wafer. A comparison of pattern sizes between the photomask and the wafer after etch reveals minimal variation, regardless of the pattern size. The slight deviation observed in the actual pattern size within the LMP, compared to the drawn size on the photomask, is attributed to over-etching of the LMP on the Si wafer. However, the actual pattern size in the LMP remains almost unchanged before and after the etch. Error bars represent mean ± standard deviation ($n = 4$)

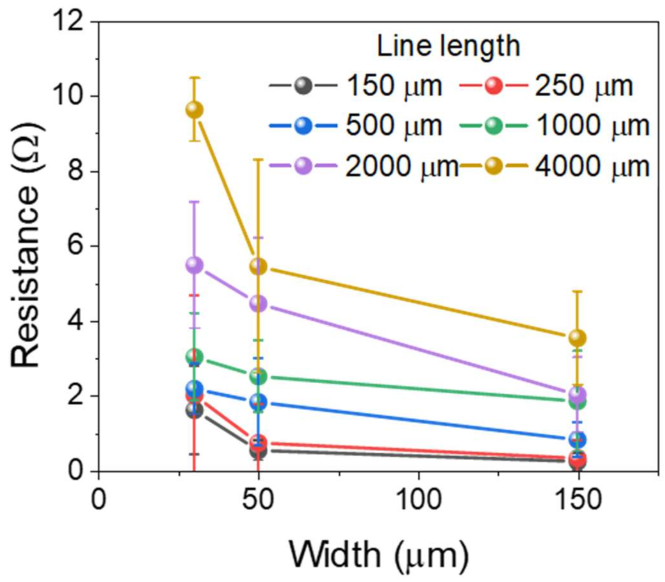

Supplementary Fig. 9 | Measured resistance of patterned LMP lines for various line widths and lengths. We evaluated the resistance of micro-patterned LMP lines at various dimensions, widths ranging from 30 μm to 150 μm and lengths from 150 μm to 4000 μm. As expected, the resistance of the LMP lines increased with length and decreased with width. Error bars represent mean ± standard deviation (n = 10)

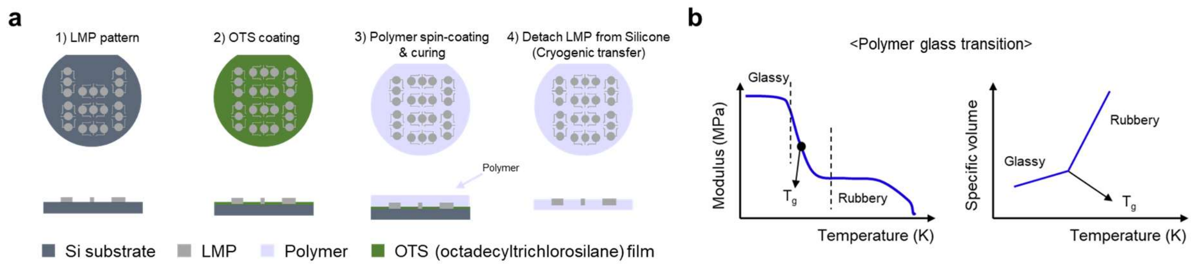

Supplementary Fig. 10 | Schematic of the cryogenic transfer process of patterned LMP onto polymer substrates. a, Schematic of cryogenic transfer process. Detailed description of the transfer process is presented in the Method section. 1) LMP patterns were delineated using photolithography and etching on a silicon wafer (donor substrate). 2) Coating octadecyltrichlorosilane (OTS) on the patterned LMP film. This process only applies to the polymers that have strong adhesion with Si substrates. 3) Spin-coating polymer on the top of the wafer and then curing. 4) Immersing in liquid nitrogen (77 K) to transfer the patterned LMP from the Si substrate to polymer substrates (acceptor substrate). **b,** Modulus and volume change of polymer after glass transition.

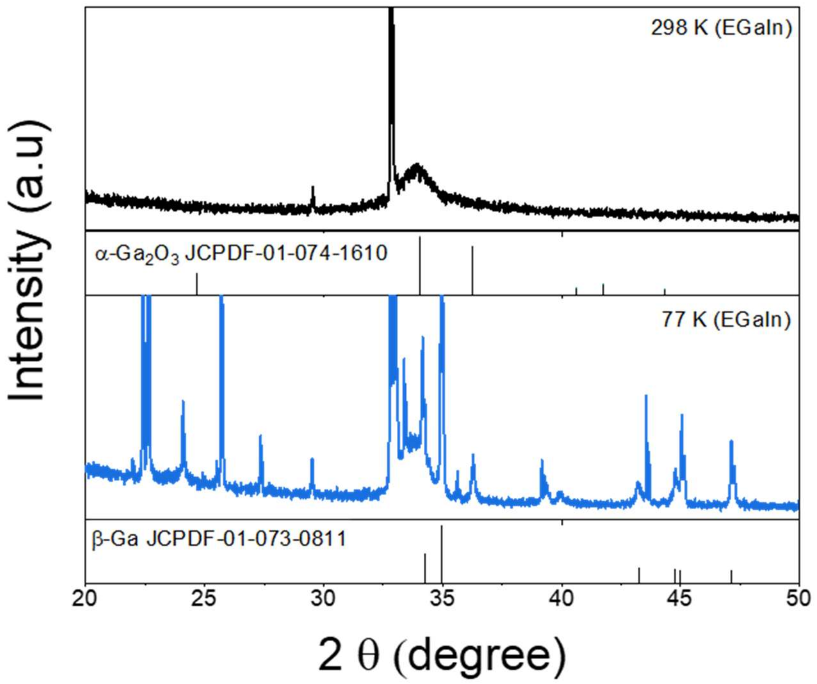

Supplementary Fig. 11 | XRD data of liquid metal at different temperatures (77 K, 298 K). At room temperature, LMP consists of a LM core and a poorly crystallized native oxide shell surrounding the LM. The (104) plane of hexagonal α-Ga_2O_3 is dominant in the native gallium oxide shell. As the temperature decreased to 77 K, the LM core crystallizes into various phases, with β-Ga being predominant.

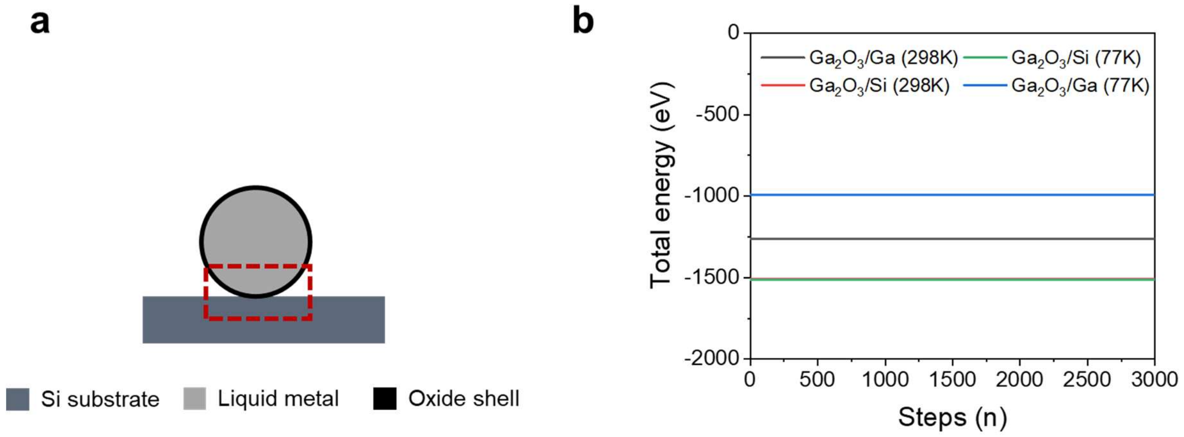

Supplementary Fig. 12 | AIMD simulation data on the binding energy between Si and LMP at different temperatures (77 K, 298 K) and various interfaces in the system. a, Schematic cross-sectional image of LMP on the Si substrate **b,** Calculated total energy for the interfaces at 298 K and 77 K (3000 steps).

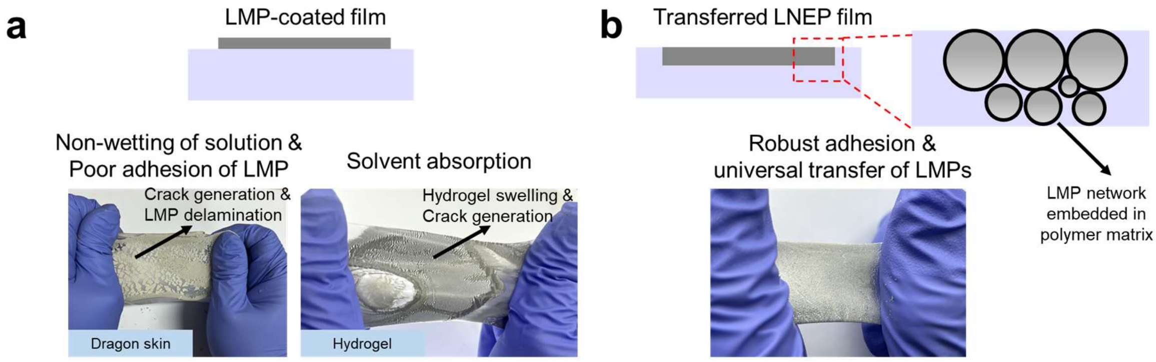

Supplementary Fig. 13 | Comparison of LMP-coated film and transferred LNEP film. a, Schematic of the cross-sectional view of LMP coated on a polymer substrate (top), along with photographs of LMP-coated dragon skin (bottom, left) and LMP-coated hydrogel (bottom, right) under strain. **b,** Schematic of the cross-sectional view of the LNEP (top) and photograph of LNEP (bottom) under strain. In the LMP coating method, cracks were easily generated, and LMP were delaminated due to the wettability of the solution on the polymer substrate and poor adhesion between LMP and the substrates. Additionally, some polymers, such as hydrogels, were swollen during the LMP coating process. In contrast, LNEP, with its universal cryogenic transfer of LMP, demonstrates robust adhesion to substrates even under strain.

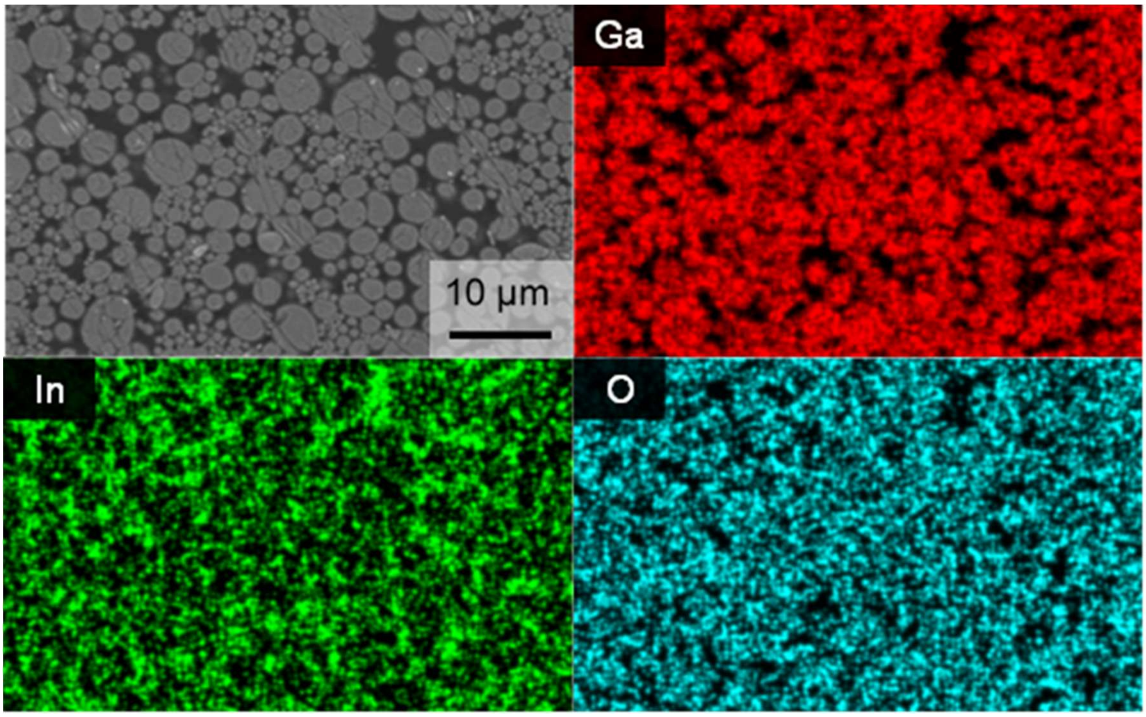

Supplementary Fig. 14 | EDS mapping image of the LNEP.

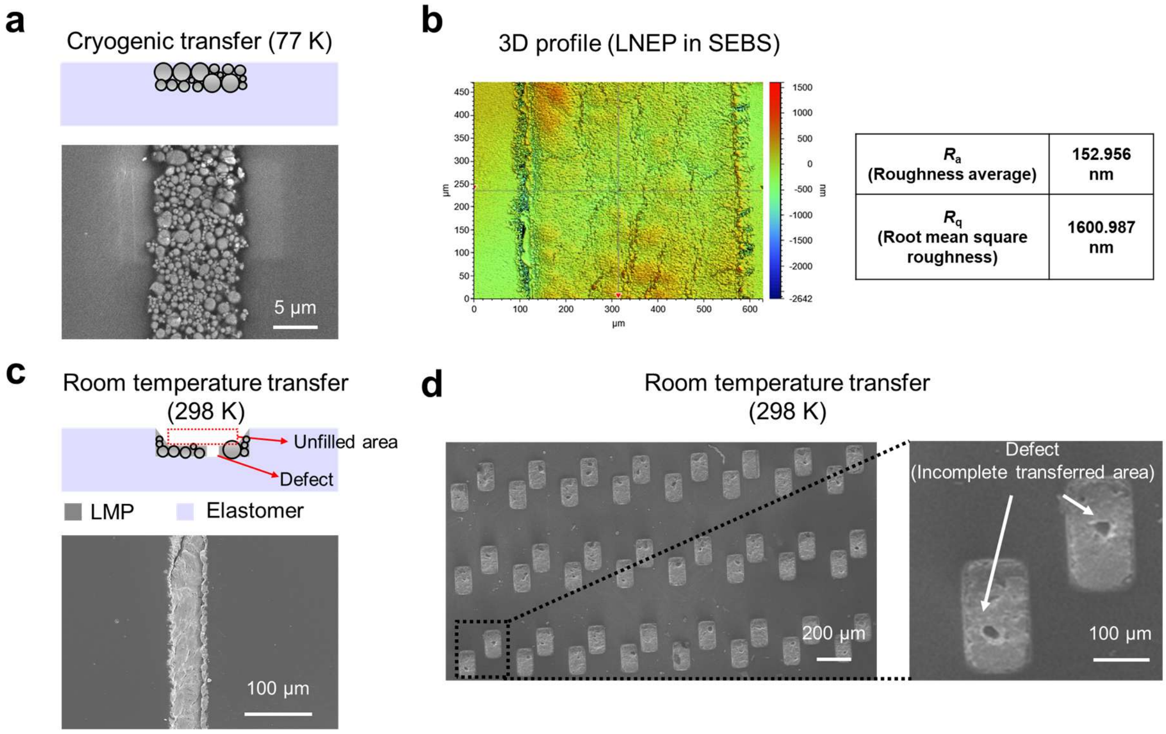

Supplementary Fig. 15 | Comparison of LMP pattern transfer at 77 K and 298 K. a, Schematic of the cross-sectional view (top) and SEM image of the top view (bottom) of LMP lines in elastomer after cryogenic transfer. **b**, 3D profile image of LMP embedded in SEBS by cryogenic transfer, with roughness average (R_a) and root mean square roughness (R_q). **c,** Schematic of the cross-sectional view (top) and SEM image of the top view (bottom) of LMP lines in elastomer after the transfer at 298 K. The transfer at 298 K results in unfilled areas and defects due to the incomplete transfer. **d**, SEM image of the top view of island LMP patterns in elastomer after the transfer at 298 K. The magnified image shows unfilled regions due to the incomplete transfer.

When the LMP patterns were transferred to the elastomer at 298 K, the patterns failed to detach completely from the Si wafer, resulting in a partial loss of LMP. Consequently, the transferred LMP patterns exhibited unfilled areas, which can sometimes disrupt the pattern. In contrast, cryogenic transfer results in entire transfer of LMP patterns. According to 3D profile analysis, the transferred LMP film shows flat morphology, which facilitates subsequent processes.

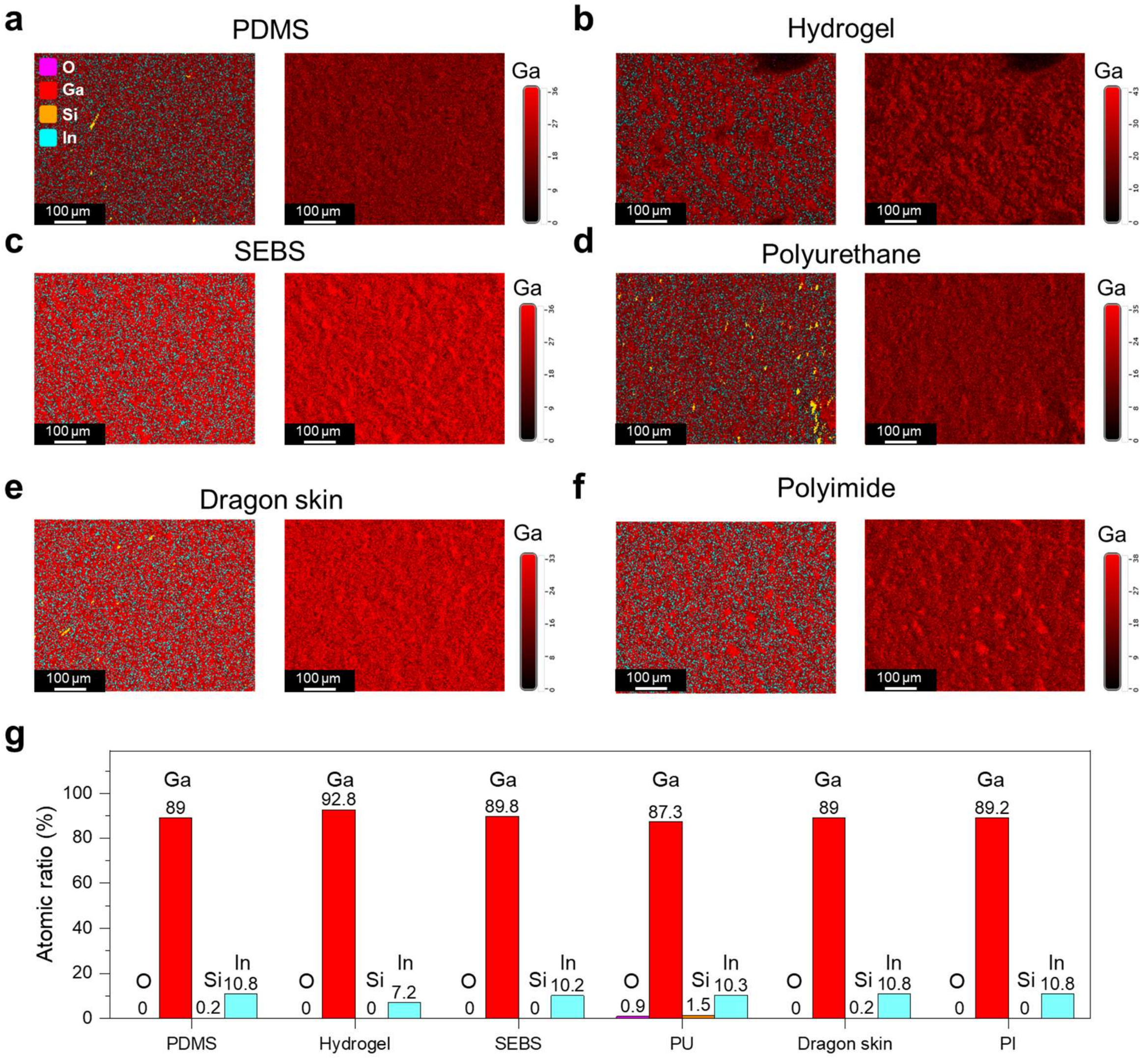

Supplementary Fig. 16 | EDS-SEM images of LMP-patterned wafer after room temperature transfer to various polymers using EGaIn liquid metal. a-f. EDS-SEM analysis of all elements (left) and Ga element (right) on the initial Si wafers (donor substrate) after transfer to PDMS (**a**), hydrogel (**b**), SEBS (**c**), polyurethane (**d**), dragon skin (**e**), polyimide substrates (**f**). Ga element remained on the Si wafer due to the incomplete transfer at 298 K. **g,** Element atomic ratios obtained by EDS analysis for a-f. The Ga content is overwhelmingly dominant in the residues. This result confirms that the LMP patterns cannot be entirely transferred to the polymer substrate at room temperature.

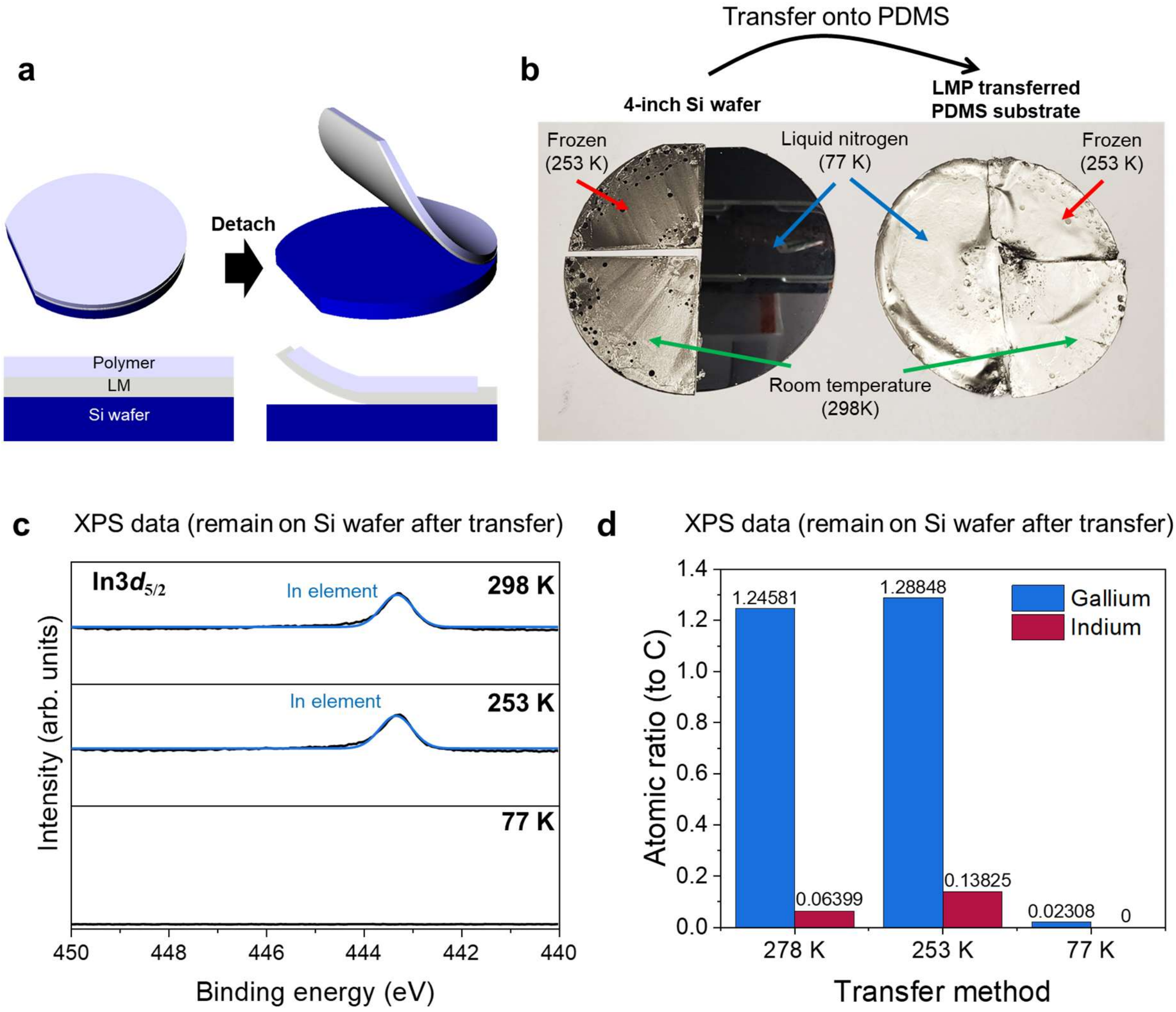

Supplementary Fig. 17 | Analysis of the residues on Si wafer after transfer to polymer substrates at various temperatures. a, Schematic of the transfer process of LMP film from the Si wafer to polymer substrates. **b**, Photographs of the initial LMP-coated Si wafer (left) and LMP-transferred PDMS (right) after transfer under different temperature conditions (77 K, 253 K, 298 K). **c,** XPS results of In3$d_{5/2}$ from Si wafers after different transfer temperatures (77 K, 253 K, 298 K). The absence of any peaks indicates that no residues remain on the Si wafer after the cryogenic transfer. **d**, XPS data of elements (Ga, In) remaining on the Si wafer surface after the transfer process at different temperatures. When detaching the LMP film from the Si wafer and transferring to the PDMS at 298 K and 253 K, the LMP film was partially transferred to the PDMS; however, some were left on the Si wafer, indicating incomplete transfer. In contrast, after cryogenic transfer, neither Ga nor In was observed on the silicon wafer, indicating complete transfer of the LMPs.

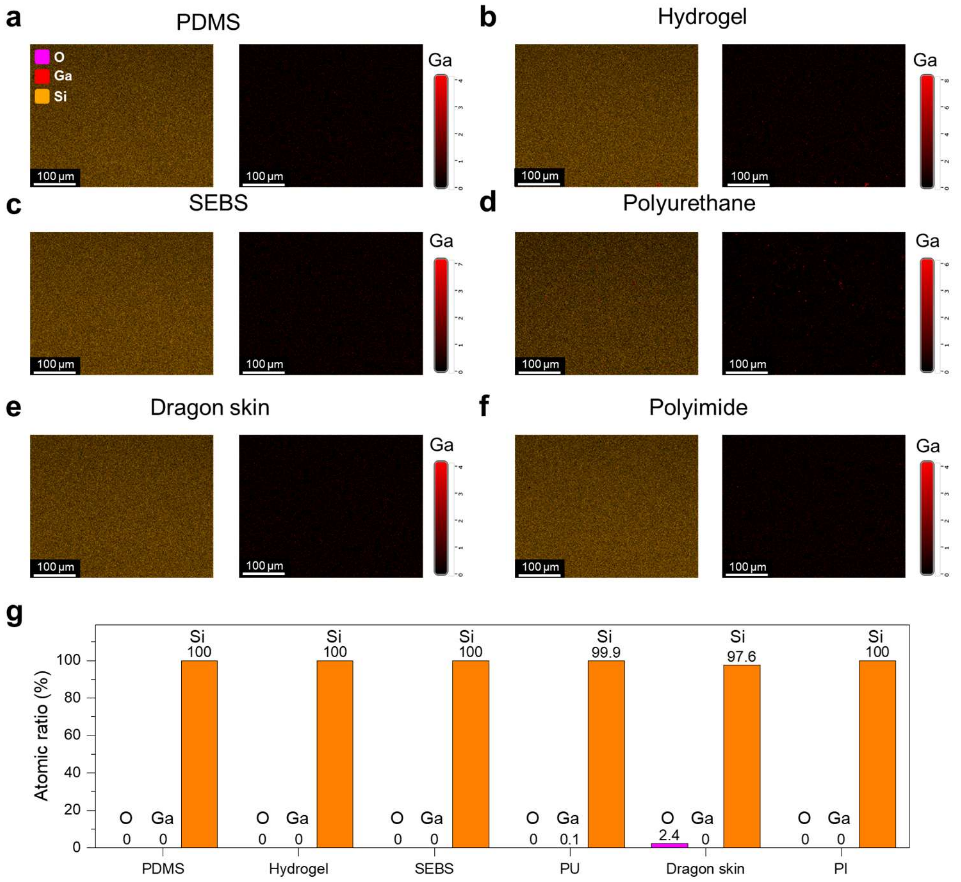

Supplementary Fig. 18 | EDS-SEM images of LMP-patterned wafer after cryogenic transfer using Ga liquid metal. a-f, EDS-SEM analysis of all elements (left) and Ga element (right) on the initial Si wafers (donor substrate) after transfer to PDMS (**a**)**,** hydrogel (**b**)**,** SEBS (**c**), polyurethane (**d**)**,** dragon skin (**e**)**,** polyimide substrates (**f**). **g,** The element atomic ratios obtained by EDS analysis for a-f. The Ga content is nearly 0%, indicating that the entire LMP patterns were transferred after the cryogenic transfer.

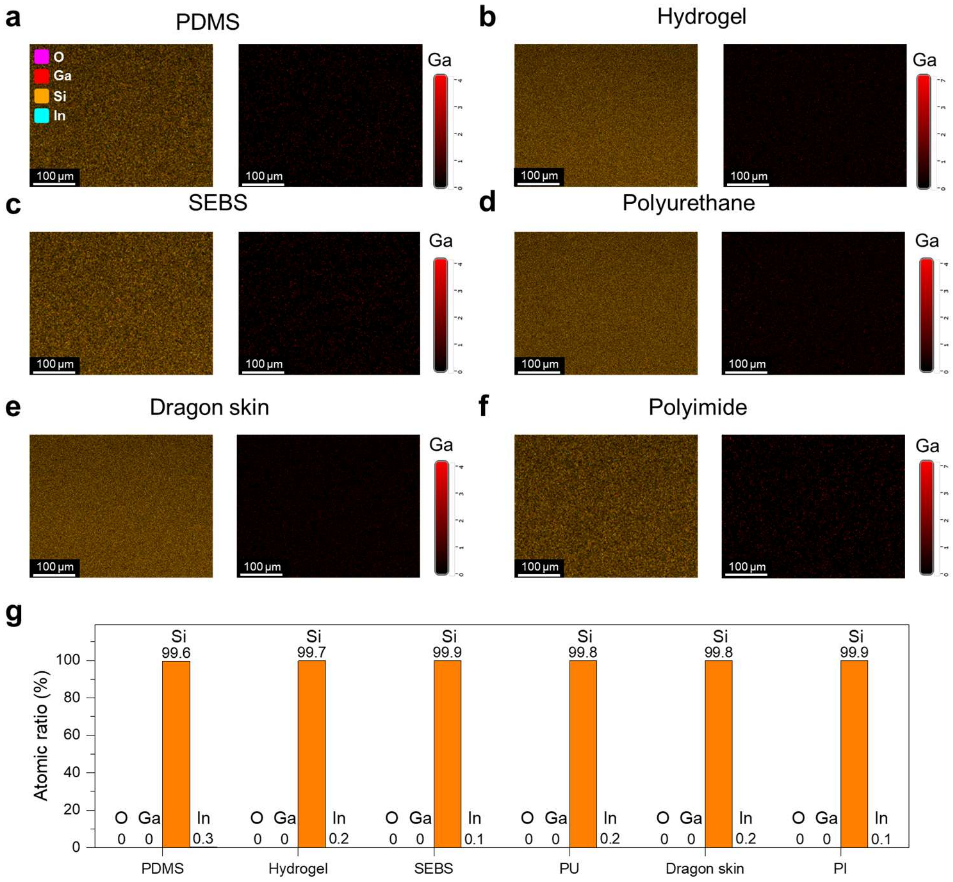

Supplementary Fig. 19 | EDS-SEM images of LMP-patterned wafer after cryogenic transfer using EGaIn liquid metal. a-f, EDS-SEM analysis of all elements (left) and Ga element (right) on the initial Si wafers (donor substrate) after transfer to PDMS (**a**), hydrogel (**b**), SEBS (**c**), polyurethane (**d**), dragon skin (**e**), polyimide substrates (**f**). **g,** The element atomic ratios obtained by EDS analysis for a-f. The Ga content is nearly 0%, indicating that the entire LMP patterns were transferred after the cryogenic transfer.

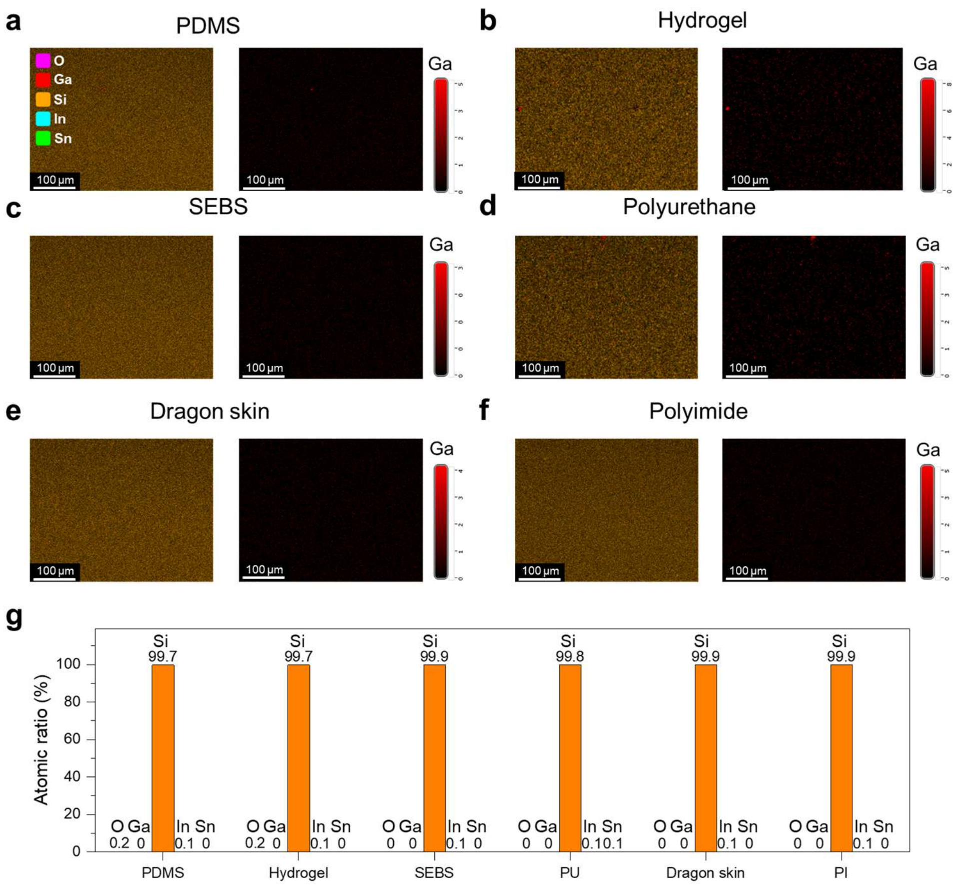

Supplementary Fig. 20 | EDS-SEM images of LMP-patterned wafer after cryogenic transfer using Galinstan liquid metal. a-f, EDS-SEM analysis of all elements (left) and Ga element (right) on the initial Si wafers (donor substrate) after transfer to PDMS (**a**), hydrogel (**b**), SEBS (**c**), polyurethane (**d**), dragon skin (**e**), polyimide substrates (**f**). **g,** The element atomic ratios obtained by EDS analysis for a-f. The Ga content is nearly 0%, indicating that the entire LMP patterns were transferred after the cryogenic transfer.

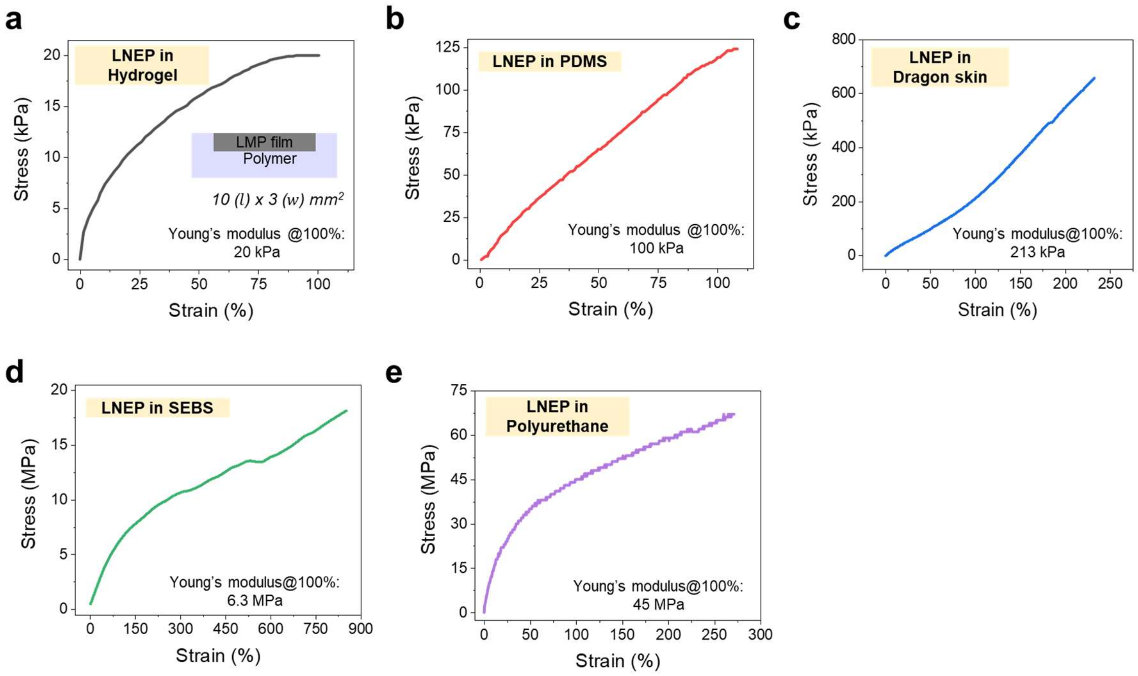

Supplementary Fig. 21 | Mechanical properties of LNEP in various polymer substrates. **a,** LNEP in hydrogel **b,** LNEP in PDMS **c,** LNEP in Dragon skin **d,** LNEP in SEBS **e,** LNEP in polyurethane.

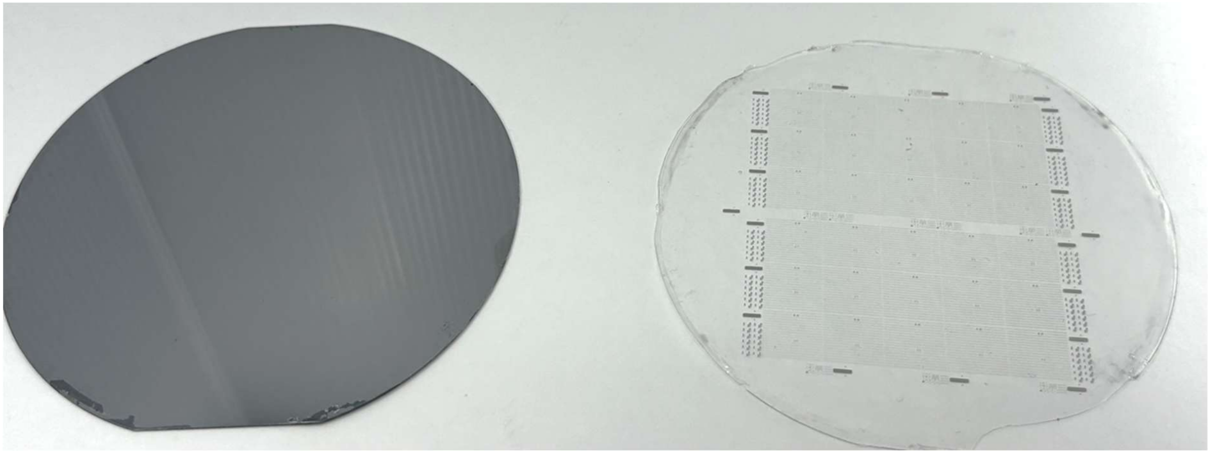

Supplementary Fig. 22 | Wafer-scale cryogenic transfer. Photograph of LMP-patterned Si wafer after cryogenic transfer (left), and transferred LMP film on the SEBS polymer (right).

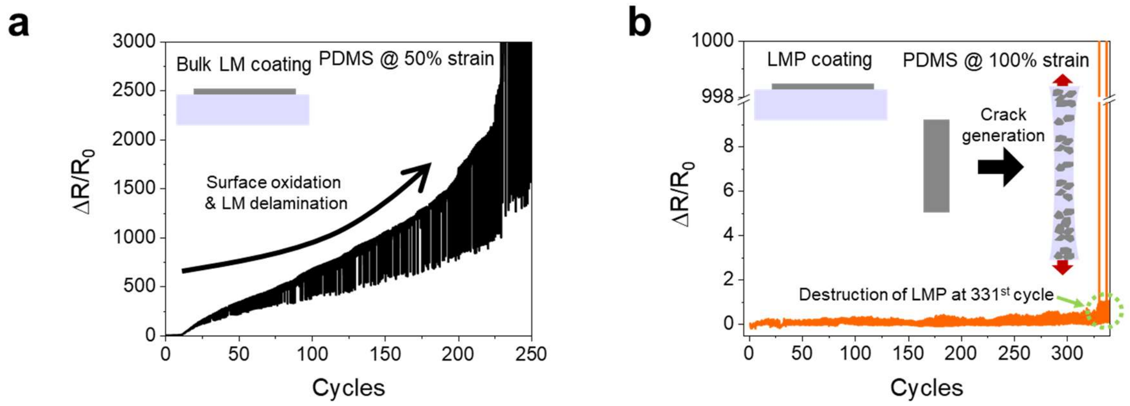

Supplementary Fig. 23 | Cyclic test of resistance change for the coated LM and LMP on PDMS. a, Cyclic test of electrical resistance change from 0% to 50% strain for bulk LM coating on PDMS. The increase in resistance change is due to surface oxidation and LM delamination. **b,** Cyclic test of electrical resistance change from 0% to 100% strain for LMP coating on PDMS. At the 331th cycles, LMP cracks were generated at 100 % strain, leading to the destruction of the electrode.

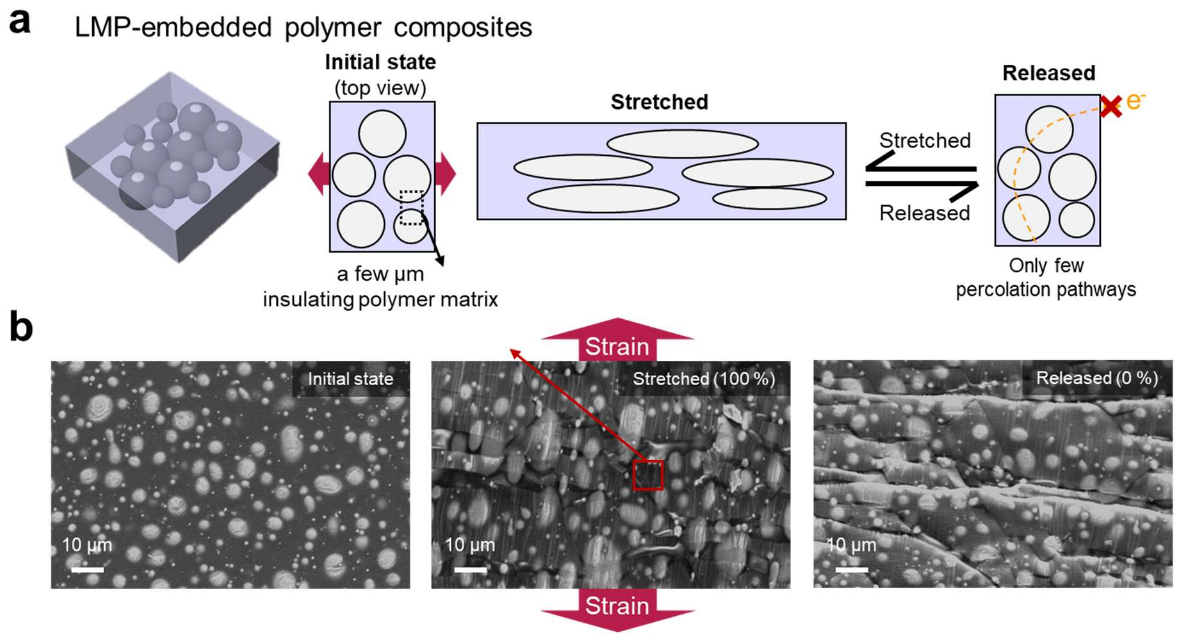

Supplementary Fig. 24 | Electromechanical properties of LMP-embedded polymer composites. a, Schematic illustrations dipict the percolation network mechanism of the LMP-embedded polymer composite before and after uniaxial stretching. **b,** SEM images show the LMP-embedded polymer composite in the initial state, 100% stretched state, and released state.

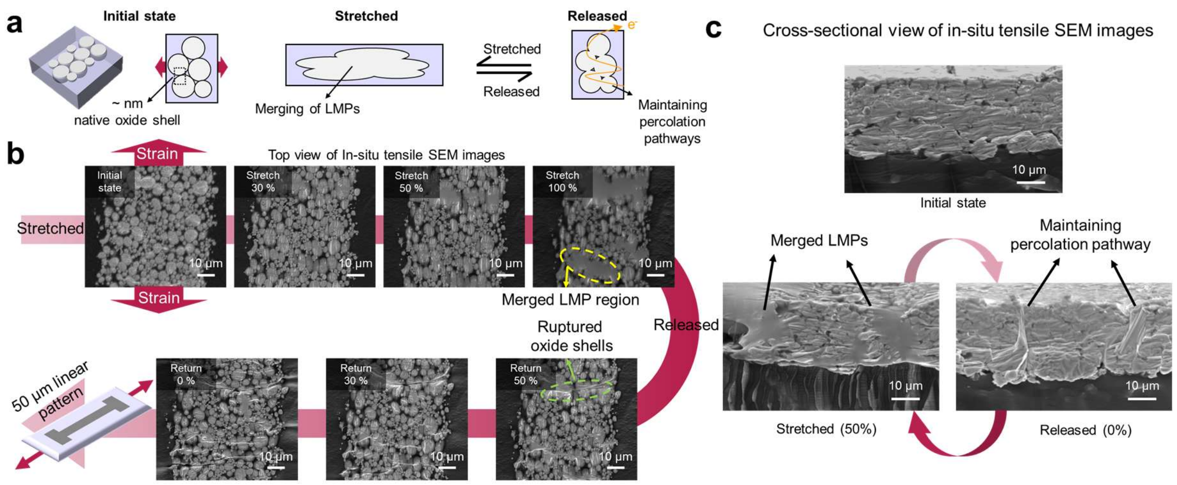

Supplementary Fig. 25 | Electromechanical properties of LNEP. a, Schematic illustrations depict the percolation network mechanism of the LNEP before and after uniaxial stretching **b,** Top-view SEM image of a 50 µm LNEP line from 0% to 100% strain and back from 100% to 0%. The merging of LMPs during stretching was observed in the yellow dotted circle in the 100% stretching image. The clustered ruptured oxide shells during the released state can be observed in the green dotted circle in the 50% return image. **c,** SEM image of the cross-sectional view of LNEP 0% to 50% strain and back from 100% to the 0%. The merging of LMPs was observed in the stretched SEM image, while the maintenance of the percolation pathway was evident in the released SEM image.

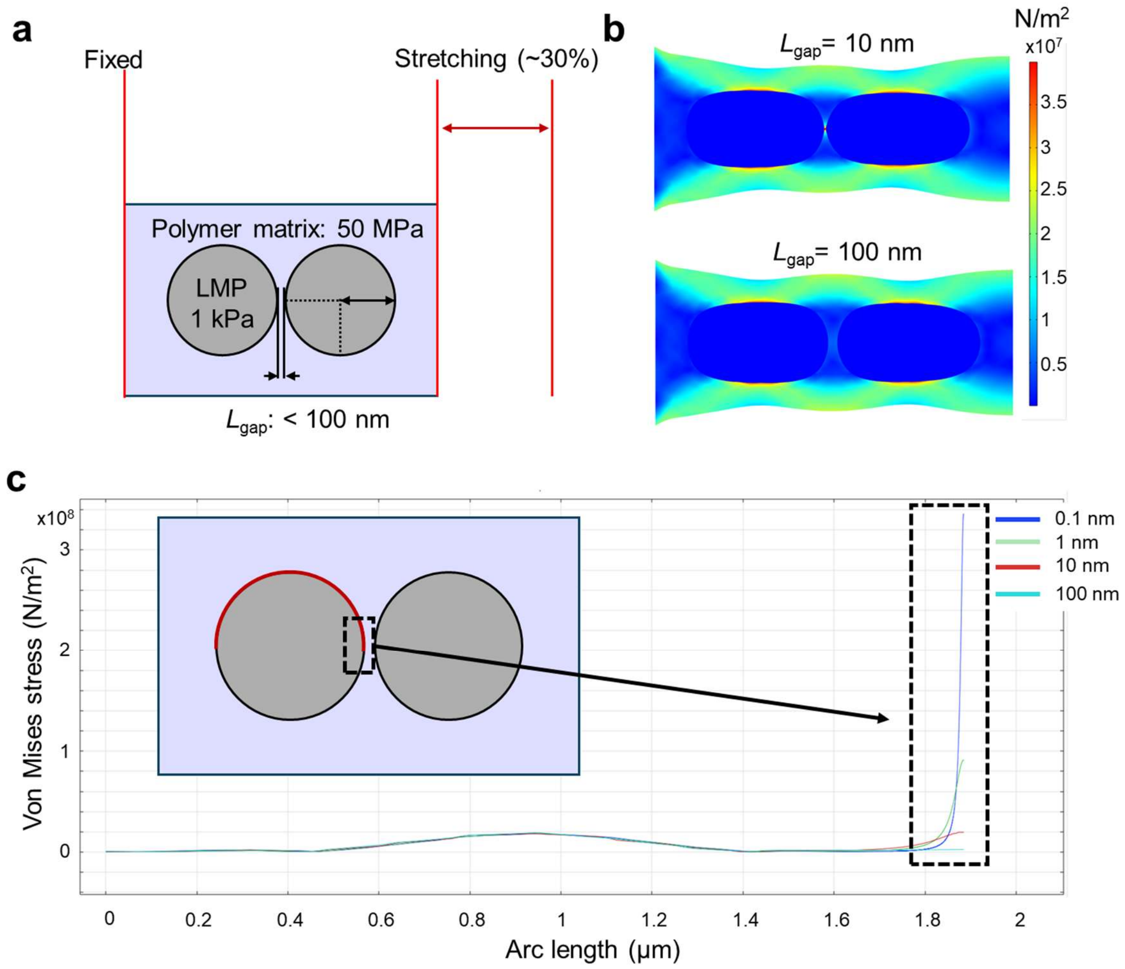

Supplementary Fig. 26 | A finite element (FE) simulation showing the stress distribution between two LMPs using COMSOL Multiphysics. a, Schematic illustration of the unit cell conditions in the FE simulation, when 30% strain was applied to the right side of the unit cell. The Young's modulus (E_o) of the polymer matrix and LMP was set to 50 MPa and 1 kPa, respectively. **b,** FE simulation images showing the stress concentration under strain for gap sizes (L_{gap}) of 10 nm (top) and 100 nm (bottom). As the L_{gap} decreases, the neck region between the LMPs becomes concentrated with stress, causing the LMP shell to break. **c,** Applied stress at the LMP shell for different L_{gap} of 0.1, 1, 10, and 100 nm. The stress at the shell increases significantly as the L_{gap} decreases.

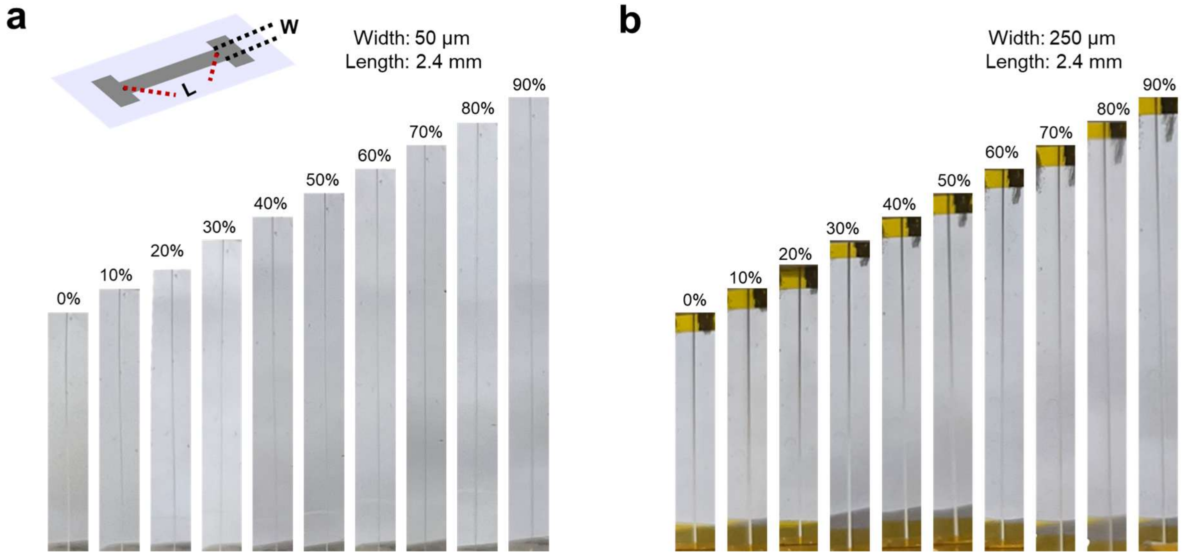

Supplementary Fig. 27 | Photographs of tensile testing of LNEP line in PDMS. Tensile tests from 0% to 90% in increments of 10% for two LNEP lines in PDMS. **a,** Schematic illustration of the LNEP lines. Photographs of tensile test of LNEP line with width 50 μm and length 2.4 mm **b,** Photographs of tensile test of LNEP line with width 250 μm and length 2.4 mm.

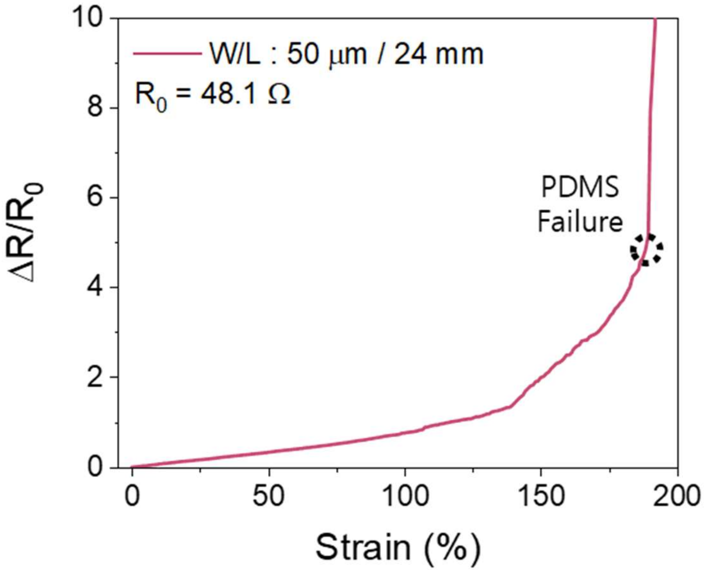

Supplementary Fig. 28 | Resistance response of LNEP line in PDMS. Up to 145% strain, the resistance of LNEP increases gradually. At 190% strain, the resistance response sharply increases due to PDMS failure observed at the dotted circle. (R_0: 48.1 Ω without strain, width: 50 μm, length: 24 mm)

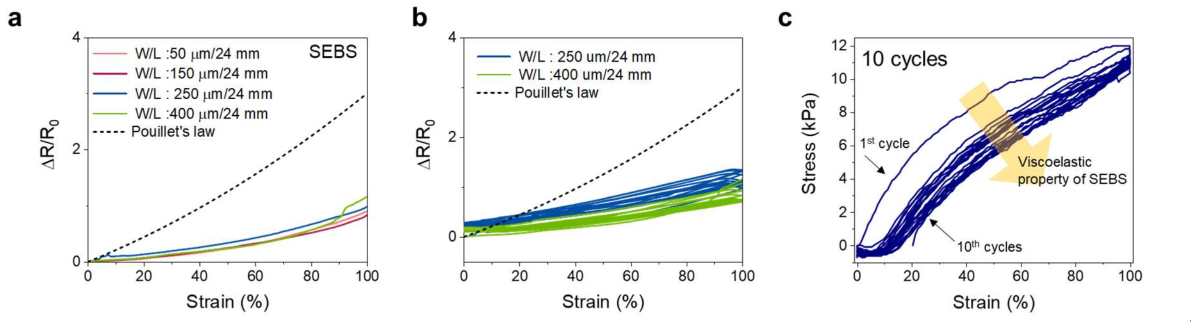

Supplementary Fig. 29 | Electrical properties of LNEP line in SEBS. a, Resistance response of LNEP lines in SEBS with different widths (50, 150, 250, 400 μm) over the same length (24 mm). **b**, Resistance response of LNEP lines in SEBS under 100% strain over 10 cycles, with widths of 250 μm, 400 μm and a length of 24 mm. **c**, Stress versus strain for the LNEP lines in SEBS under 10 cycles of repeated 100% strain.

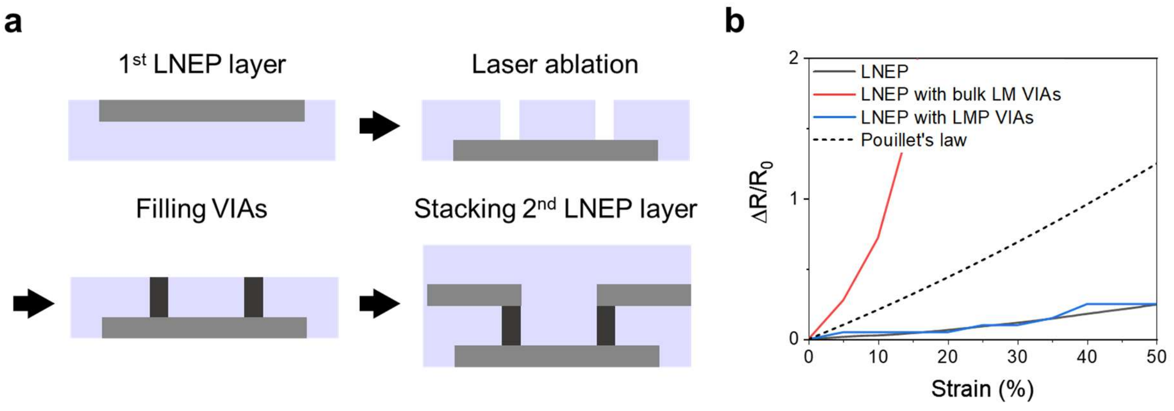

Supplementary Fig. 30 | Stretchable multilayer LNEP using stretchable VIAs. a, Fabrication process of multilayer LNEP. The backside of the 1st LNEP layer was drilled using a CO_2 laser, and the created VIAs were filled with LMP ink. Subsequently, the 2nd LNEP layer was stacked on top of the 1st LNEP layer. **b,** Resistance response of multilayer LNEP with stretchable VIAs. The black line represents the single-layer LNEP, the red line represents the multilayer LNEP with bulk LM VIAs, and the blue line represents the multilayer LNEP with LMP VIAs. The LMP VIAs exhibited strain-insensitive properties compared to the bulk LM VIAs.

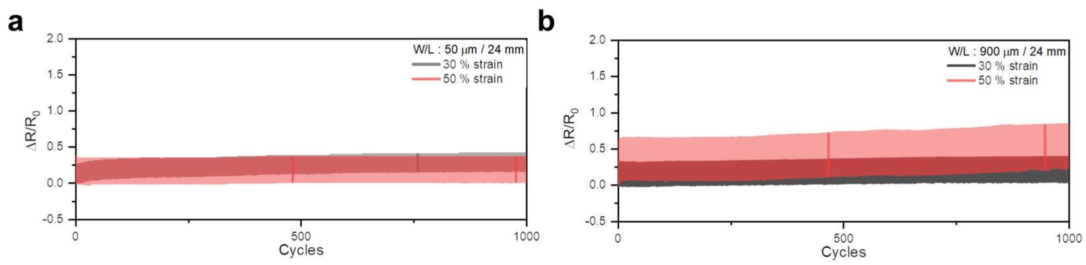

Supplementary Fig. 31 | Long-term stability of LNEP line in PDMS under iterative strain (30%, 50%) up to 1,000 cycles. a, b $\Delta R/R_0$ of LNEP lines in PDMS under 30% and 50% strain during 1,000 cycles, with widths of 50 µm **(a)**, 900 µm **(b)** and a length of 24 mm.

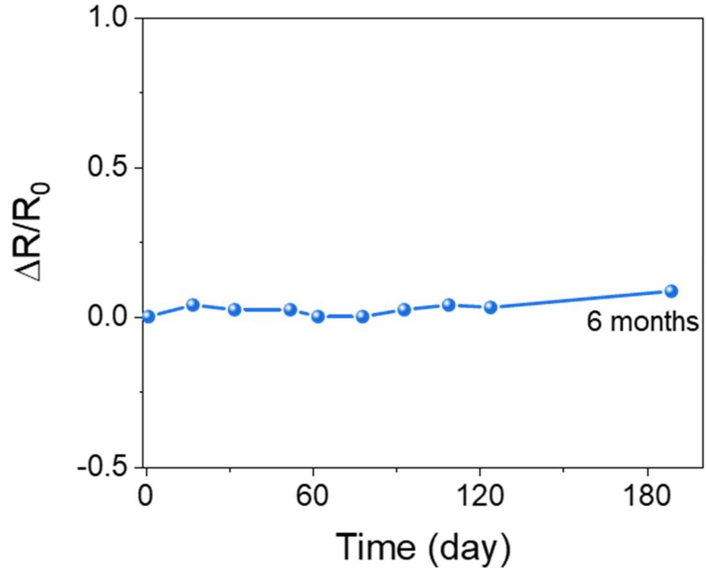

Supplementary Fig. 32 | Long-term resistance stability of the LNEP electrode evaluated over a half-year at room temperature.

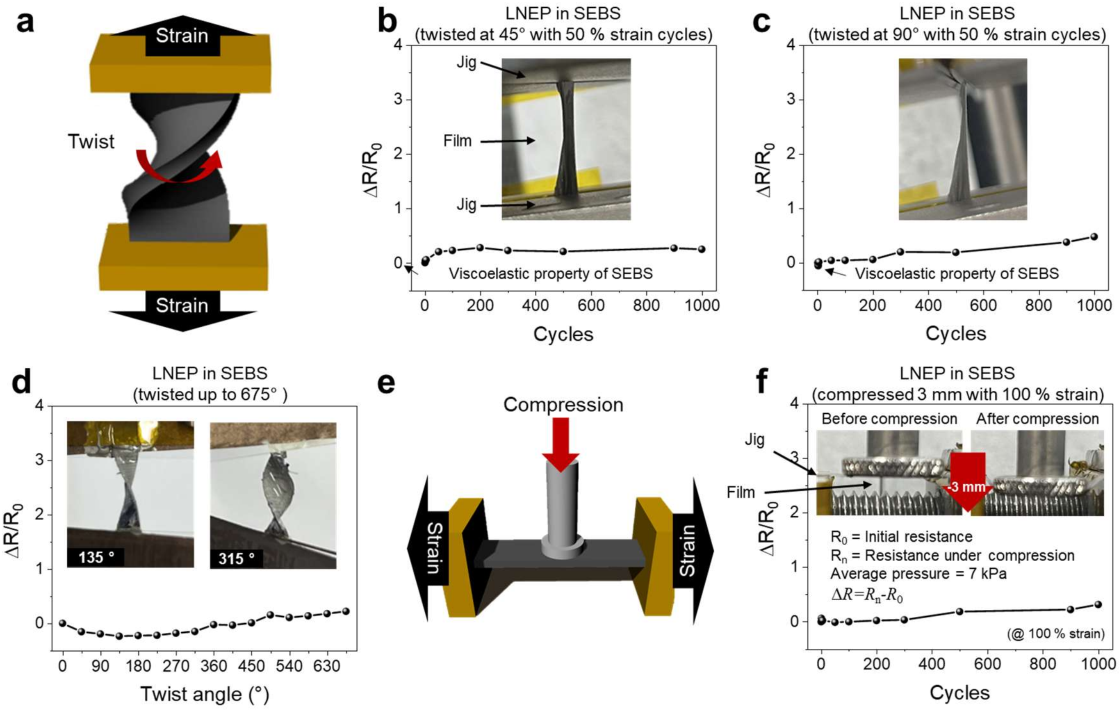

Supplementary Fig. 33 | Electrical stability of LNEP in SEBS under various mechanical deformations. a, Schematic illustration of LNEP in SEBS under twisting deformation with stretching. **b-c**, $\Delta R/R_0$ of LNEP in SEBS with 50% strain under twisted angle of 45° (**b**) and 90° (**c**) over 1000 cycles. **d**, $\Delta R/R_0$ of LNEP in SEBS under twisting deformation according to the twisting angle (up to 675°). **e**, Schematic illustration of LNEP in SEBS under compression deformation with stretching. **f**, $\Delta R/R_0$ of LNEP in SEBS compressed by 3 mm over 1000 cycles while stretched up to 100% strain (average pressure: 7 kPa).

When LNEP in SEBS is twisted by 45° and 90°, the relative change in resistance slightly increases during the initial 10 cycles due to the viscoelasticity of the SEBS polymer. As the number of stretching cycles progresses, the relative change in resistance of LNEP in SEBS at both 45° and 90° becomes stable. Even when twisted incrementally up to 675° in the twisting mode, it exhibits stable electrical resistance changes.

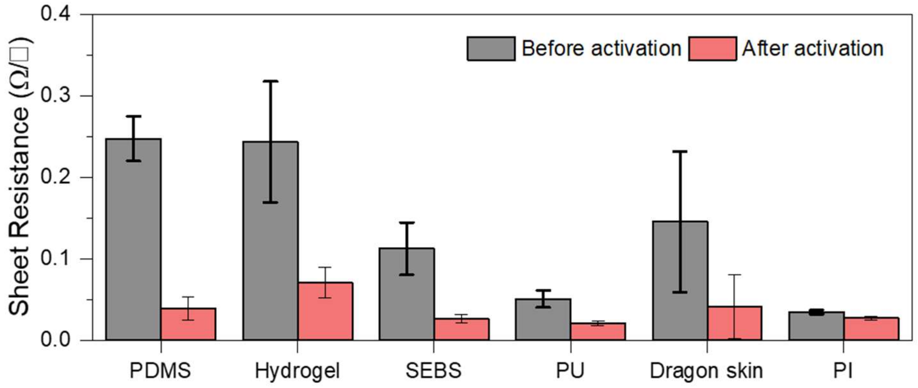

Supplementary Fig. 34 | The sheet resistance of LNEP in 6 different polymers before and after activation by strain.

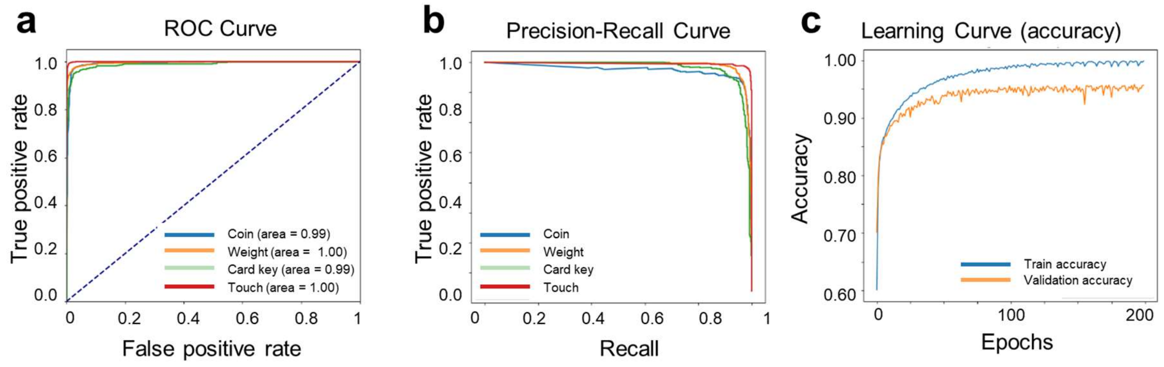

Supplementary Fig. 35 | Performance evaluation of the neural network model for 4 categories (touch, card key, weight, coin). a, Receiver operating characteristic curves (ROC) for each category **b,** Precision-recall curves (PRC) of model performances for each category **c,** Learning curves showing the progression of training and validation accuracy.

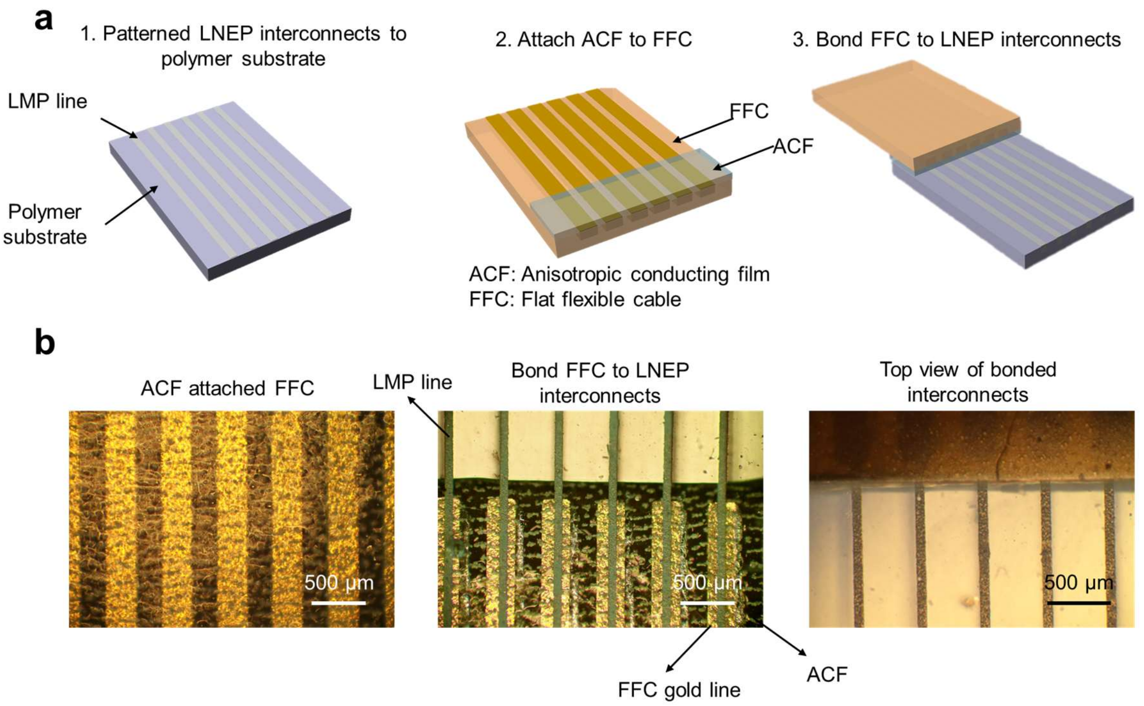

Supplementary Fig. 36 | Bonding FFC to patterned LNEP interconnects. a, Schematic illustration of the FFC bonding process. **b,** Optical microscope image of the FFC bonding process.

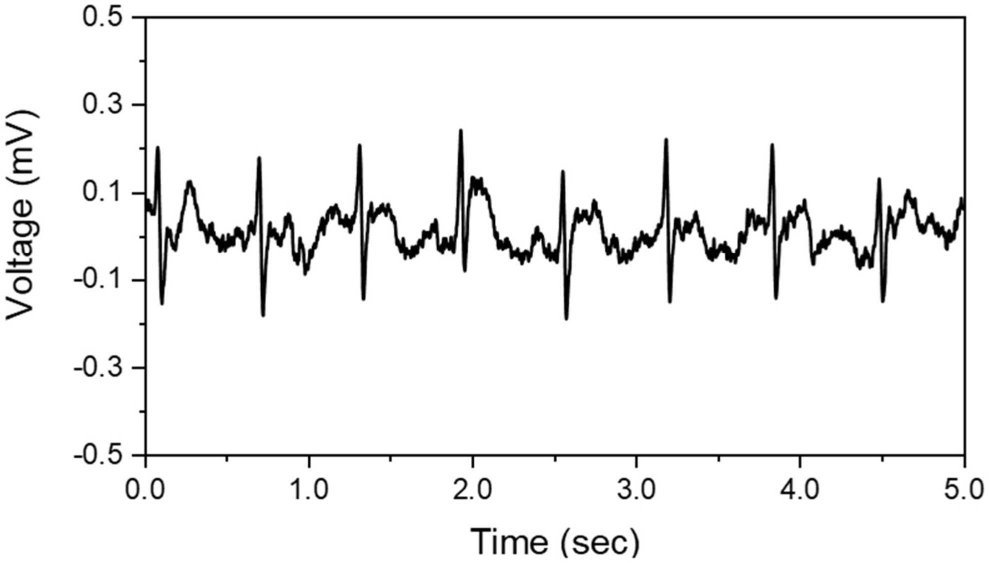

Supplementary Fig. 37 | Real-time ECG signal collected with LNEP wearable multifunctional sensor.

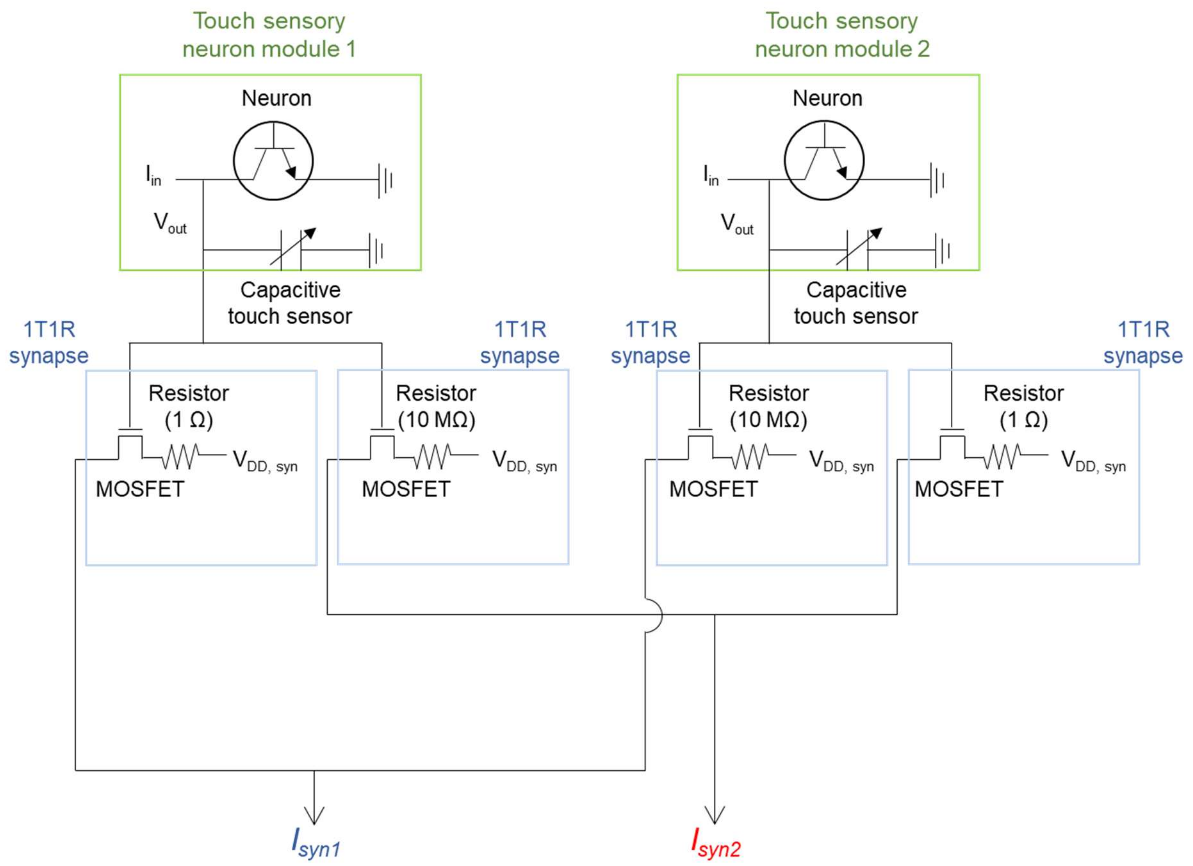

Supplementary Fig. 38 | Circuit diagram of the stretchable touch-sensory neuromorphic circuit configuration (2 neurons × 4 synapses). Each synapse consists of a transistor (1T) and a resistor (1R). The 1R modulates the synaptic weight by adjusting its resistance.

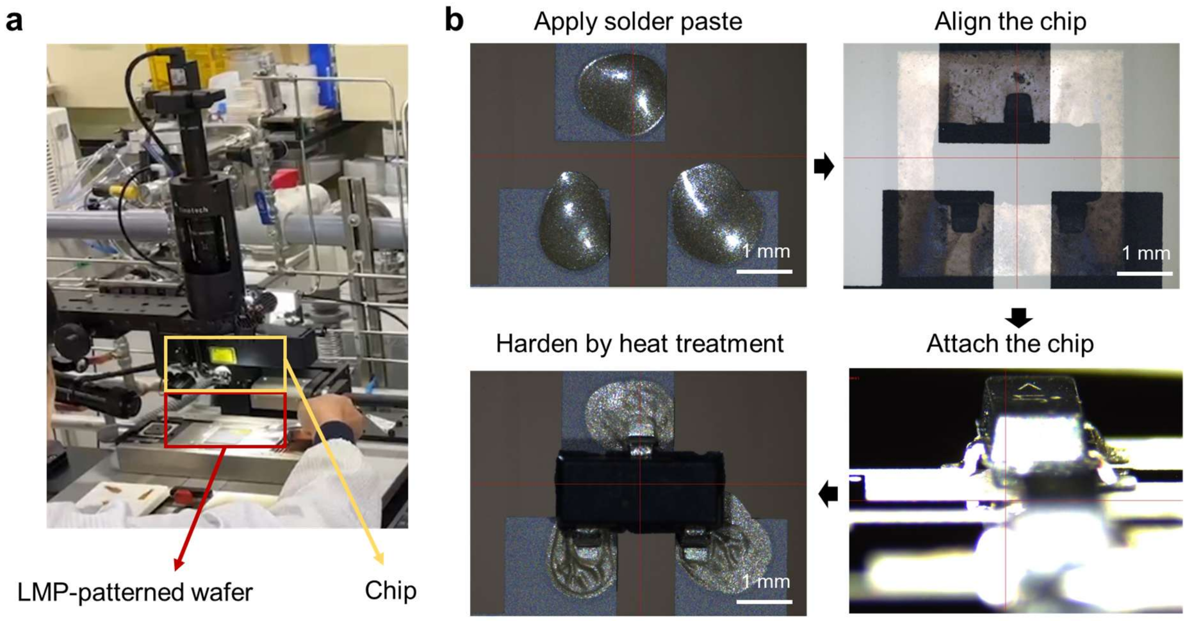

Supplementary Fig. 39 | Chip bonding process using the chip bonder. a, Photograph of the chip bonder. **b,** Optical microscope images of the sequential chip bonding process.

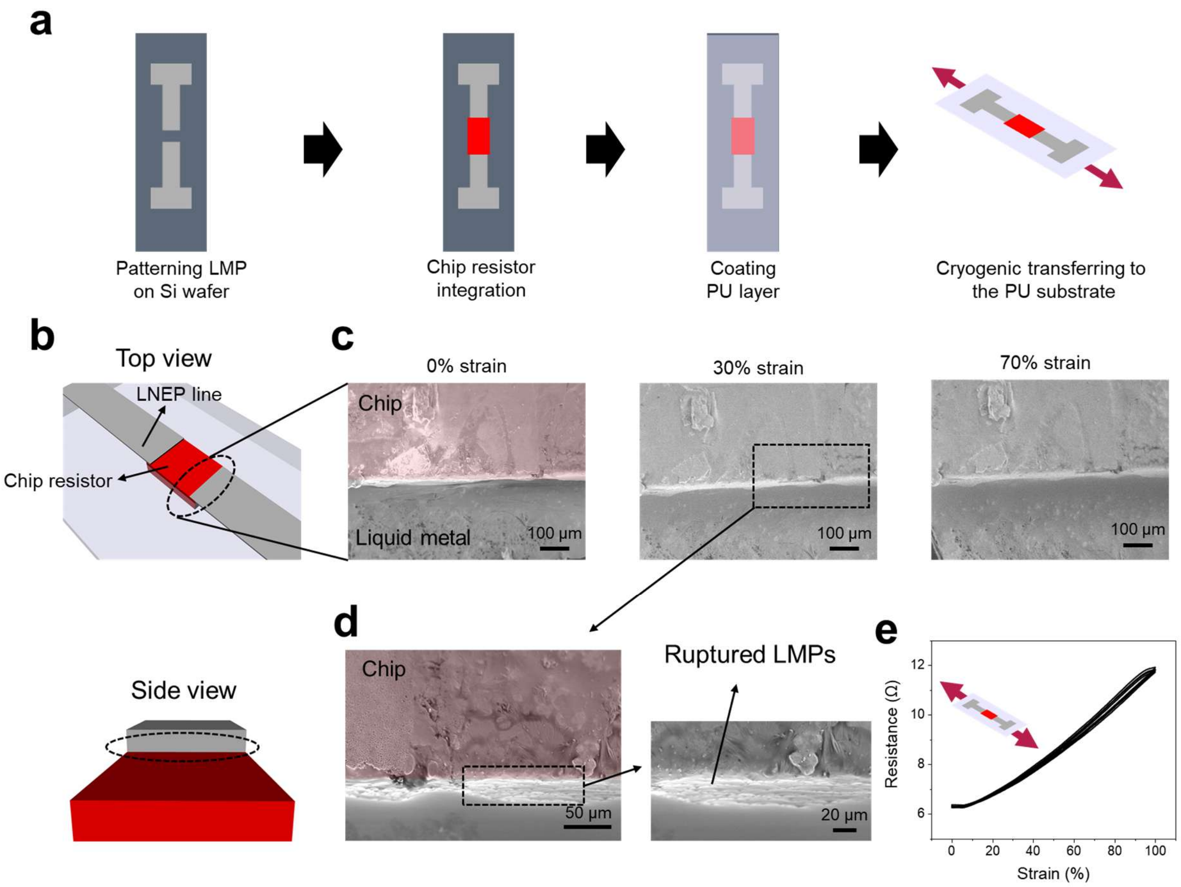

Supplementary Fig. 40 | Electromechanical properties of LNEP bonded with a chip resistor. a, Schematic illustrations of the chip bonding process. **b,** Schematic illustrations of the chip resistor bonded with the LNEP **c,** SEM images showing the interface between the chip and liquid metal according to the strain (0%, 30%, 70%). The transferred LNEP exhibited robust adhesion with the chips even under 70% strain. **d,** Magnified SEM image of the interface under 30% strain, showing that the chips maintain a stable electrical connection with the LNEP due to the rupturing of LMPs. **e,** The resistance response of LNEP with the chip resistor under strain. The resistance increased by 87% at 100% strain, which is comparable to 90% increase observed in LNEP at 100% strain. In bulk conductors, the resistance increases by 300% at 100% strain, as described by Pouillet's law [$\Delta R/R_0 = (1+\varepsilon)^2-1$].

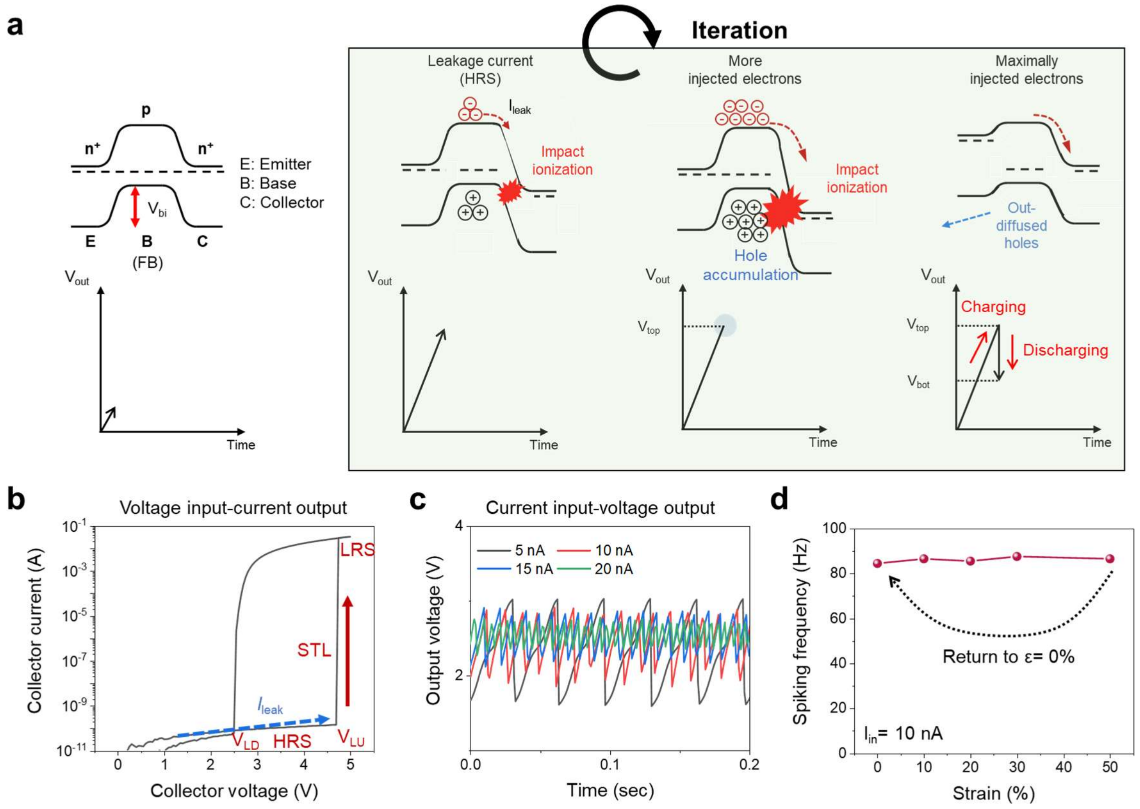

Supplementary Fig. 41 | Working mechanism of the artificial neuron device. a, Step-by-step energy band diagrams and schematic plots of oscillating V_{out} vs. time for one cycle of the neuron device. **b,** Measured collector current (I_C)vs. collector voltage (V_C) of the neuron device. Latch-up voltage (V_{LU}) and latch-down voltage (V_{LD}) were observed and threshold switching driven by a single transistor latch (STL) phenomenon was also found. While high-resistance state (HRS) was dominated by leakage current (I_{leak}), low-resistance state (LRS) was governed by on-state I_C. **c,** Measured spiking V_{out} vs. time for various input currents (I_{in}). Spiking frequency (*f)* was modulated by I_{in} and tended to be higher as I_{in} increased. **d,** Spiking frequency of the neuron device according to the strain (0% to 50%). The spiking characteristic was insensitive to the strain.

The artificial neuron used is a bipolar junction transistor (BJT) in a base-open configuration. In this setup, the open base acts as a floating body (FB). The spiking operation in the artificial neuron relies on the STL phenomenon driven by impact ionization (II), which involves an abrupt transition from a HRS to a LRS. When V_C exceeds a certain threshold corresponding to V_{LU}, a significant I_C flows abruptly, triggering the instant firing of the neuron device. In contrast, as long as V_C is applied to the artificial neuron device, the LRS with a high level of I_C is sustained, and II is continuously enabled, resulting in the continuous accumulation of generated

holes in the FB. However, when V_C decreases below another threshold corresponding to V_{LD}, the I_C suddenly drops to I_{leak}, completing one cycle of firing in the neuron device. This abrupt transition from LRS to HRS is attributed to the out-diffusion of the accumulated holes from the FB to the emitter. The accumulated holes in the FB significantly lower the built-in potential (V_{bi}) between the emitter and the base, which flattens the energy band.

Similarly, when a constant input current (I_{in}) is applied to the collector instead of V_C, the artificial neuron device also generates an oscillating output voltage (V_{out}) corresponding to the spiking signal. The V_{out} waveform resembles a wedge shape, with a gradual incremental slope (charging) followed by an abrupt decremental slope (discharging). The applied I_{in} lowers the energy band, triggering II near the collector, and resulting in the creation of electron-hole pairs. The generated holes accumulate in the FB, enabling the charging process, while the generated electrons contribute to I_{leak}, accelerating the II process. When the holes charge the FB, causing the V_{bi} to lower and flatten, they suddenly escape from the FB, enabling the discharging process.

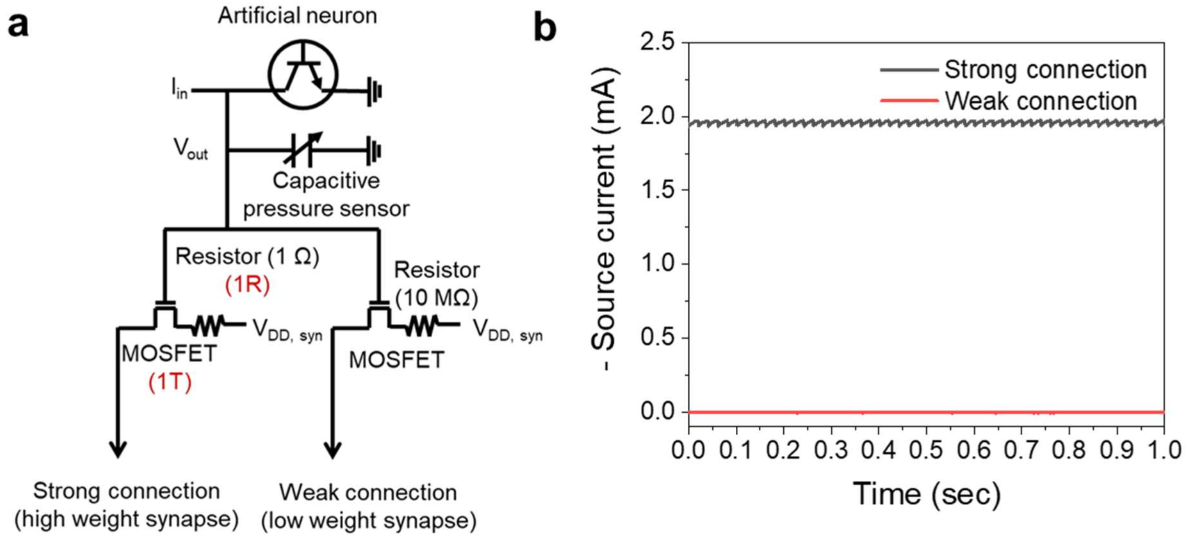

Supplementary Fig. 42 | Representation of strong and weak synaptic connections using binary weights with a load resistor in 1T1R synapse. a, Circuit diagram illustrating the strong and weak connections. **b,** Synaptic currents corresponding to the strong and weak connections.

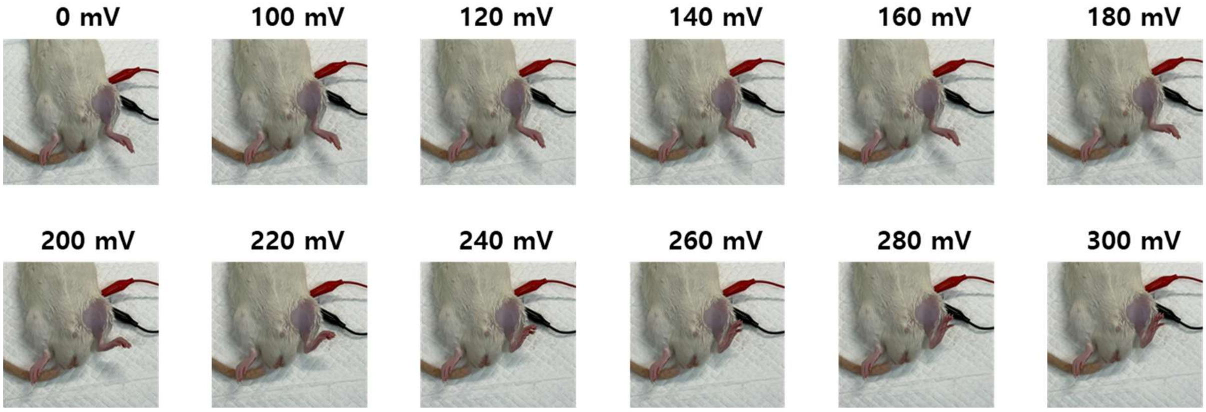

Supplementary Fig. 43 | Photographs of leg movement as increasing stimulation voltage.

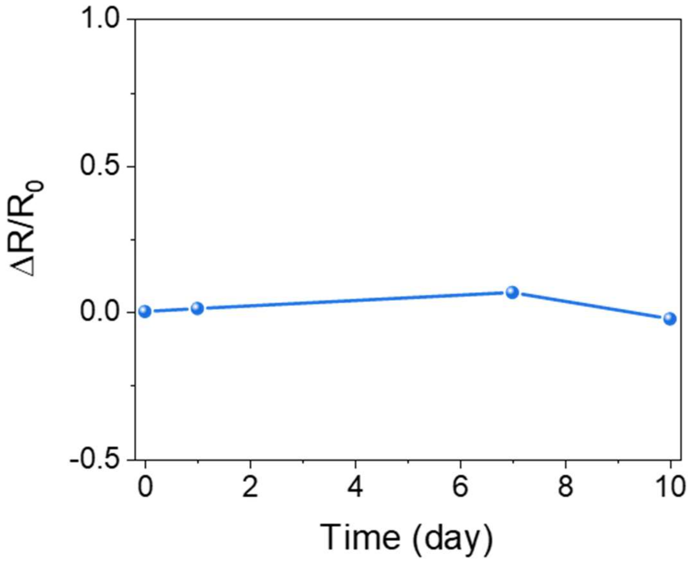

Supplementary Fig. 44 | In-vitro aging test for evaluating long-term stability of LNEP-based device in PBS solution at 50 °C.

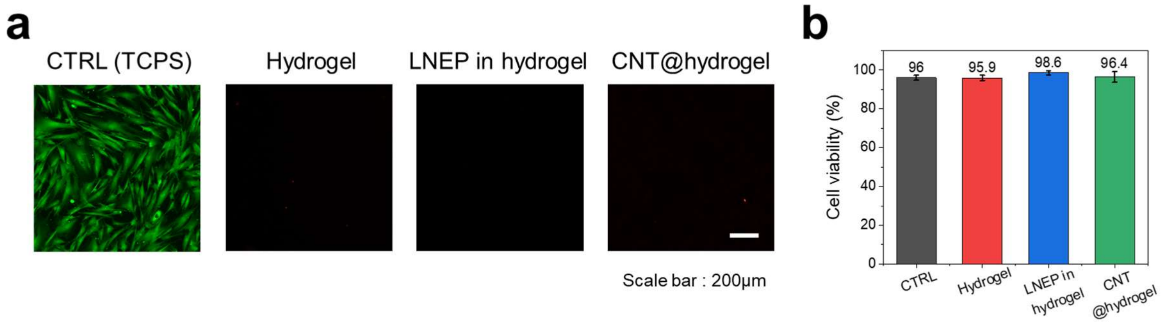

Supplementary Fig. 45 | In vitro biocompatibility test. a, Live/Dead assay images of each component of the fabricated LNEP-based in vivo device. Since no dead cells were observed in the hydrogel, LNEP in hydrogel, and CNT@hydrogel, each component is demonstrated to be noncytotoxic. **b,** Cell viability of each component of the fabricated LNEP-based in vivo device. (*n* = 3 independent material samples)

The biocompatibility test of the LNEP in hydrogel demonstrated a cell viability of 98.6%, which is comparable to cells cultured in fresh medium on a standard tissue culture plate (TCPS). This high biocompatibility is attributed to the encapsulation of LMPs within the hydrogel matrix. However, to address potential risks of deterioration, biocompatible CNT@hydrogel was employed as the working electrode by coating it onto the LNEP.

References

1. Kim, M. G., Brown, D. K. & Brand, O. Nanofabrication for all-soft and high-density electronic devices based on liquid metal. *Nat. Commun.* **11**, 1002 (2020).
2. Zhuang, Q. N. *et al.* Wafer-patterned, permeable, and stretchable liquid metal microelectrodes for implantable bioelectronics with chronic biocompatibility. *Sci. Adv.* **9**, eadg8602 (2023).
3. Johnston, L., Zhang, Y., Park, H. & Dickey, M. D. Intermetallic wetting enabled high resolution liquid metal patterning for 3D and flexible electronics. *J. Mater. Chem. C* **10**, 921–931 (2022).
4. Li, G., Wu, X. & Lee, D.-W. Selectively plated stretchable liquid metal wires for transparent electronics. *Sens. Actuators B Chem.* **221**, 1114–1119 (2015).
5. Park, C. W. *et al.* Photolithography-based patterning of liquid metal interconnects for monolithically integrated stretchable circuits. *ACS Appl. Mater. Interfaces* **8**, 15459-15465 (2016).
6. Lee, G. H. *et al.* Large-area photo-patterning of initially conductive EGaIn particle-assembled film for soft electronics. *Mater. Today* **67**, 84-94 (2023).
7. Gozen, B. A., Tabatabai, A., Ozdoganlar, O. B. & Majidi, C. High-density soft-matter electronics with micron-scale line width. *Adv. Mater.* **26**, 5211-5216 (2014).
8. Kim, M. G., Alrowais, H., Pavlidis, S. & Brand, O. Size-scalable and high-density liquid-metal-based soft electronic passive components and circuits using soft lithography. *Adv. Funct. Mater.* **27**, 1604466 (2017).
9. Gao, Y. J. *et al.* Wearable microfluidic diaphragm pressure sensor for health and tactile touch monitoring. *Adv. Mater.* **29**, 1701985 (2017).
10. Lin, Y. L. *et al.* Vacuum filling of complex microchannels with liquid metal. *Lab Chip,* **17**, 3043-3050 (2017).
11. Wang, Q., Yu, Y., Yang, J. & Liu, J. Fast fabrication of flexible functional circuits based on liquid metal dual-trans printing. *Adv. Mater.* **27**, 7109–7116 (2015).
12. Boley, J. W., White, E. L., Chiu, G. T. C. & Kramer, R. K. Direct writing of gallium-indium alloy for stretchable electronics. *Adv. Funct. Mater.* **24**, 3501-3507 (2014).
13. Lee, G. H. *et al.* Rapid meniscus-guided printing of stable semi-solid-state liquid metal microgranular-particle for soft electronics. *Nat. Commun.* **13**, 2643 (2022).
14. Tang, L., Wu, Y., Wu, Z. & Li, Y. Large-scale fabrication of highly elastic conductors

on a broad range of surfaces. *ACS Appl. Mater. Interfaces* **11**, 7138–7147 (2019).

15. Sun, Y.-C., Boero, G. & Brugger, J. Stretchable conductors fabricated by stencil lithography and centrifugal force-assisted patterning of liquid metal. *ACS Appl. Electron. Mater.* **3**, 5423–5432 (2021).
16. Zhu, H., Cheng, S., Yu, R., Jiang, H. & Lu, Y. Fully solution processed liquid metal features as highly conductive and ultrastretchable conductors. *NPJ Flex. Electron.* **5**, 25 (2021).
17. Xuan Li et al., High-resolution liquid metal–based stretchable electronics enabled by colloidal self-assembly and microtransfer printing. *Sci. Adv.* **11**, eadw3044 (2025).
18. J. Ma, Z. Sa, H. Zhang, J. Feng, J. Wen, S. Wang, Y. Tian, Microconfined Assembly of High-Resolution and Mechanically Robust EGaIn Liquid Metal Stretchable Electrodes for Wearable Electronic Systems. *Adv. Sci.* **11**, 2402818 (2024).
19. J. Ma, Z. Liu, and P. Zhang, Precisely Patterning Liquid Metal Microfibers Through Electrohydrodynamic Printing for Soft Conductive Composites and Electronics. *Adv. Mater.* **37**, 2507646 (2025).
20. J. Pyeon, H. Lee, W. Choe, S. Park, and H. Kim, Versatile Liquid Metal Composite Inks for Printable, Durable, and Ultra-Stretchable Electronics. *Small* **21**, 2501829 (2025).
21. Pan, C. F. *et al.* Visually imperceptible liquid-metal circuits for transparent, stretchable electronics with direct laser writing. *Adv. Mater.* **30**, 1706937 (2018).
22. Zhao, R., Zhang, Q., Liu, T., Wang, C. & Liu, J. A fast and cost-effective transfer printing of liquid metal inks for three-dimensional wiring in flexible electronics. *ACS Appl. Mater. Interfaces* **12**, 36723–36730 (2020).
23. Guo, R., Liu, J., Sheng, L., Wang, Q. & Liu, J. One-step liquid metal transfer printing: toward fabrication of flexible electronics on wide range of substrates. *Adv. Mater. Technol.* **3**, 1800265 (2018).
24. Guo, R., Zhang, W., Yang, Y., Wang, Q. & Liu, J. Thermal transfer-enabled rapid printing of liquid metal circuits on multiple substrates. *ACS Appl. Mater. Interfaces* **14**, 37028–37038 (2022).
25. Zhou, Z., Wang, W., Wang, Y., Wu, Y. & Liu, J. Hybrid transfer printing of liquid metals and allied inks for rapid fabrication of multifunctional soft electronics. *ACS Appl. Mater. Interfaces* **16**, 25589–25599 (2024).
26. Zalewski, K., Chyłek, Z. & Trzciński, W. A. A review of polysiloxanes in terms of their application in explosives. *Polymers* **13**, 1080 (2021).

27. Taşdemir, M., Şenaslan, F. & Çelik, A. Investigation of corrosion and thermal behavior of PU–PDMS-coated AISI 316L. *e-Polymers* **21**, 355–365 (2021).
28. Mohd Ibrahim, N. F., Ooi, T. C., Ismail, A. F. & Hassan, H. Surface roughness effect on optical loss in waveguide using isotropically induced crosslink network of siloxane–polyimide copolymer. *J. Appl. Polym. Sci.* **137**, 49554 (2020).
29. Gupta, P., Bera, M. & Maji, P. K. Nanotailoring of sepiolite clay with poly[styrene-b-(ethylene-co-butylene)-b-styrene]: structure–property correlation. *Polym. Adv. Technol.* **28**, 1428–1437 (2017).
30. Hua, Q., Chen, Y., Li, X., Zhang, Y. & Wang, J. Harnessing the synergistic power of lignin–Ecoflex blends for enhanced performance in food packaging. *Chem. Eng. J.* **499**, 156139 (2024).
31. Vaucher, J., Bousquet, C., Leterrier, Y. & Månson, J.-A. E. Recycling of bottle grade PET: influence of HDPE contamination on the microstructure and mechanical performance of 3D printed parts. *Polymers* **14**, 5507 (2022).
32. Mersiowsky, I., Weller, M. & Ejlertsson, J. Fate of plasticised PVC products under landfill conditions: a laboratory-scale landfill simulation reactor study. *Water Res.* **35**, 3063–3070 (2001).
33. Calleja, G., Pérez, E., Ben Dhia, M., Laurent, H. & Vigier, G. Where is the glass transition temperature of poly(tetrafluoroethylene)? A new approach by dynamic rheometry and mechanical tests. *Eur. Poly*m. J. **49**, 2214–2222 (2013).
34. Yang, Q., Liu, W., Li, J., Zhang, J. & Liu, B. The glass transition temperature measurements of polyethylene: determined by using molecular dynamic method. *RSC Adv.* **6**, 12053–12060 (2016).
35. Yang, C. & Takahashi, I. Broadening, no broadening and narrowing of glass transition of supported polystyrene ultrathin films emerging under ultraslow temperature variations. *Polym. J.* **43**, 390–397 (2011).
36. Mohammadi, M. & Davoodi, J. The glass transition temperature of PMMA: a molecular dynamics study and comparison of various determination methods. *Eur. Polym. J.* **91**, 121–133 (2017).
37. Han, J. K. *et al.* artificial olfactory neuron for an in-sensor neuromorphic nose. *Adv. Sci.* **9**, 2106017 (2022).
