# Supplementary material for: Universal cryogenic transfer of liquid metal particles in polymers for wafer-scale stretchable integrated electronics
Source: Nat Commun. 2026 Feb 26;17:3248. doi: 10.1038/s41467-026-70101-2 (PMC13061968; doi:10.1038/s41467-026-70101-2)
Supplement: Supplementary file 2 — Description of Additional Supplementary Information [file 41467_2026_70101_MOESM2_ESM.pdf]

Title: Supplementary Video 1

Description: Volumetric expansion of LM in liquid nitrogen
