# Supplementary material for: Universal cryogenic transfer of liquid metal particles in polymers for wafer-scale stretchable integrated electronics
Source: Nat Commun. 2026 Feb 26;17:3248. doi: 10.1038/s41467-026-70101-2 (PMC13061968; doi:10.1038/s41467-026-70101-2)
Supplement: Supplementary file 5 — Transparent Peer Review file [file 41467_2026_70101_MOESM5_ESM.pdf]

nature portfolio

Peer Review File

Universal Cryogenic Transfer of Liquid Metal Particles in Polymers for Wafer-Scale Stretchable Integrated Electronics

Corresponding Author: Professor Steve Park

This manuscript has been previously reviewed at another journal. This document only contains information relating to versions considered at Nature Communications.

This file contains all reviewer reports in order by version, followed by all author rebuttals in order by version.

Version 0:

Reviewer comments:

Reviewer #1

(Remarks to the Author)
Accept as it is.

Reviewer #2

(Remarks to the Author)
The manuscript by Park et al. presents a high-resolution strategy for transferring patterned liquid metal (LM) particles by integrating top-down etching-based photolithography with a cryogenic transfer process onto diverse polymer substrates. This method demonstrates significant versatility for the large-area fabrication of stretchable integrated electronics. The authors further showcase the broad applicability of their large-area LM nanoparticle electrode patterns (LNEPs) in wearable sensors, neuromorphic circuits, and in vivo devices. The authors have addressed the reviewers' comments satisfactorily. I believe the quality of the work now meets the high standards of Nature Communications and therefore recommend it for publication.

Open Access This Peer Review File is licensed under a Creative Commons Attribution 4.0 International License, which permits use, sharing, adaptation, distribution and reproduction in any medium or format, as long as you give appropriate credit to the original author(s) and the source, provide a link to the Creative Commons license, and indicate if changes were made.
In cases where reviewers are anonymous, credit should be given to 'Anonymous Referee' and the source.
The images or other third party material in this Peer Review File are included in the article's Creative Commons license, unless indicated otherwise in a credit line to the material. If material is not included in the article's Creative Commons license and your intended use is not permitted by statutory regulation or exceeds the permitted use, you will need to obtain permission directly from the copyright holder.
To view a copy of this license, visit https://creativecommons.org/licenses/by/4.0/
